# The highly and perpetually upregulated thyroglobulin gene is a hallmark of functional thyrocytes

**DOI:** 10.3389/fcell.2023.1265407

**Published:** 2023-10-04

**Authors:** Simon Ullrich, Susanne Leidescher, Yana Feodorova, Katharina Thanisch, Jean-Baptiste Fini, Bernd Kaspers, Frank Weber, Boyka Markova, Dagmar Führer, Mirian Romitti, Stefan Krebs, Helmut Blum, Heinrich Leonhardt, Sabine Costagliola, Heike Heuer, Irina Solovei

**Affiliations:** ^1^ Biocenter, Ludwig Maximilians University Munich, Munich, Germany; ^2^ Department of Medical Biology, Medical University of Plovdiv, Division of Molecular and Regenerative Medicine, Research Institute at Medical University of Plovdiv, Plovdiv, Bulgaria; ^3^ Département Adaptations du Vivant (AVIV), Physiologie Moléculaire et Adaptation (PhyMA UMR 7221 CNRS), Muséum National d’Histoire Naturelle, CNRS, CP 32, Paris, France; ^4^ Department for Veterinary Sciences, Ludwig Maximilians University Munich, Planegg, Germany; ^5^ Department of General, Visceral and Transplantation Surgery, Section of Endocrine Surgery, University Duisburg-Essen, University Hospital Essen, Essen, Germany; ^6^ Department of Endocrinology, Diabetes and Metabolism, University Duisburg-Essen, University Hospital Essen, Essen, Germany; ^7^ IRIBHM ULB, Brussels, Belgium; ^8^ Laboratory for Functional Genome Analysis (LAFUGA), Gene Center, Ludwig Maximilians University Munich, Munich, Germany

**Keywords:** thyroglobulin gene, transcription loop, transcription, gene upregulation, thyroid hormones

## Abstract

Abnormalities are indispensable for studying normal biological processes and mechanisms. In the present work, we draw attention to the remarkable phenomenon of a perpetually and robustly upregulated gene, the thyroglobulin gene (*Tg*). The gene is expressed in the thyroid gland and, as it has been recently demonstrated, forms so-called transcription loops, easily observable by light microscopy. Using this feature, we show that *Tg* is expressed at a high level from the moment a thyroid cell acquires its identity and both alleles remain highly active over the entire life of the cell, i.e., for months or years depending on the species. We demonstrate that this high upregulation is characteristic of thyroglobulin genes in all major vertebrate groups. We provide evidence that *Tg* is not influenced by the thyroid hormone status, does not oscillate round the clock and is expressed during both the exocrine and endocrine phases of thyrocyte activity. We conclude that the thyroglobulin gene represents a unique and valuable model to study the maintenance of a high transcriptional upregulation.

## Introduction

**FIGURE 1:**
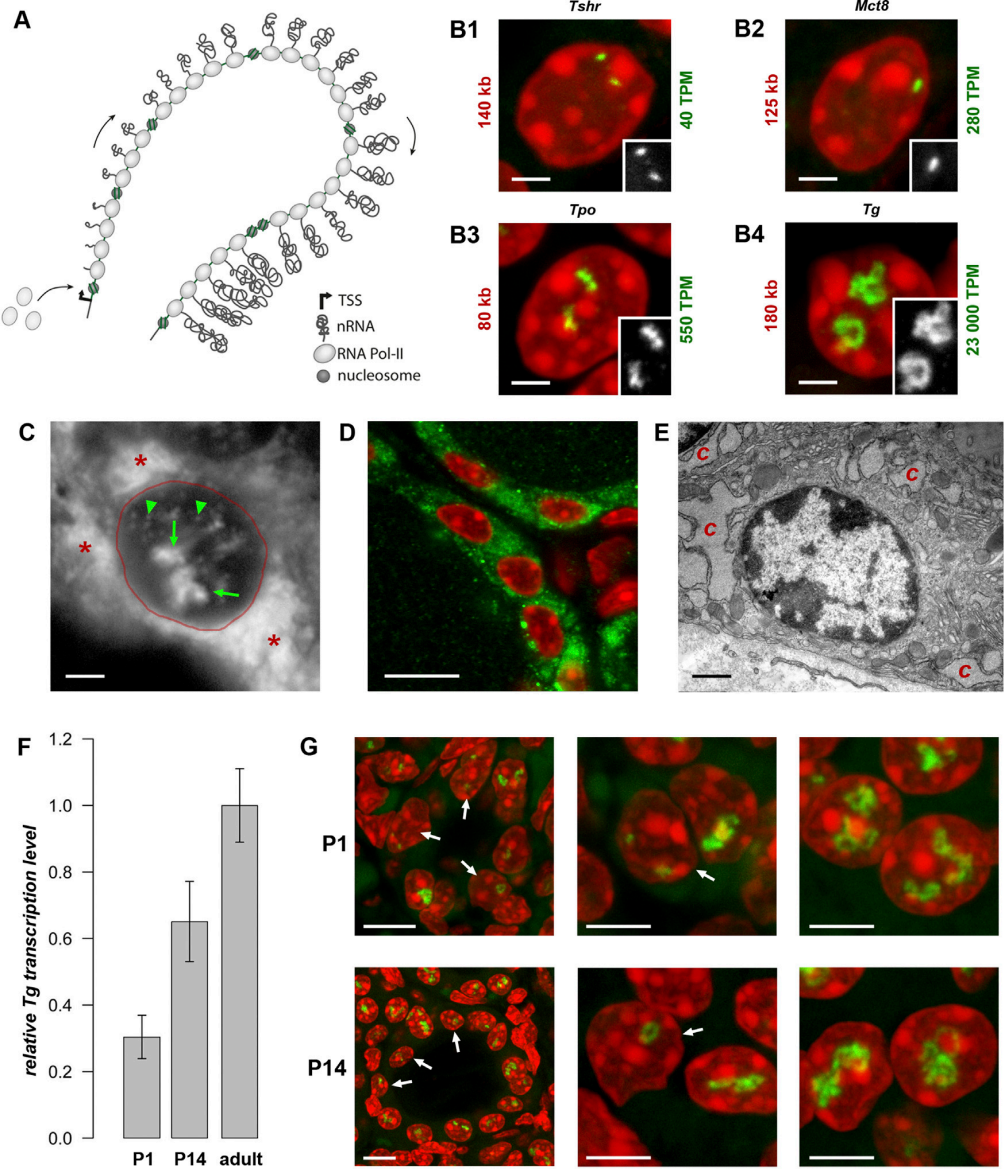
Transcription loops. **(A)** Schematics of a transcription loop (TL) formed by RNAPIIs moving along a gene and carrying nascent RNA transcripts. **(B)** Visualization of four thyroid-specific genes by RNA-FISH. In contrast to the lowly expressed genes *Tshr* and *Mct8* **(B1, B2)**, the other two genes, *Tpo* and *Tg* **(B3, B4)**, exhibit larger TLs with sizes correlated to their expression levels. Length and expression level of the genes are indicated on the left and right of the panels, respectively. For better comparison, TLs are shown as grey scale images in the insertions. Note, that in case of the *Mct8* gene located on X chromosome, there is only one signal, because the tissue originated from a male mouse. For the number of analysed nuclei, see Table 1. **(C)** RNA-FISH with an oligoprobe for *Tg* mRNA labels TLs (*green arrows*), single mRNAs in the nucleoplasm (*green arrowheads*) and in the cytoplasm (*red asterisk*). **(D,E)** Immunostaining of TG (*green*) highlights the cytoplasm of thyrocytes **(D)**, which is densely packed with remarkably hypertrophic cisterns **(C)** of endoplasmic reticulum **(E)**. **(F)** Histogram showing comparative *Tg* transcript levels in thyroids of young mice (P1 and P14) in comparison to adult mice. Since thyroid tissue includes 40% of non-thyrocyte cells, as well as chunks of practically inseparable parathyroid gland, the qPCR values were normalized to the transcription level of the thyroid specific transcription factor Pax8 to avoid erroneous results. Error bars are SDM, three biological replicates were analyzed per developmental stage The observed changes were not significant (*p* > 0.05) as determined by one-way ANOVA with Tukey’s multiple comparisons *post hoc*. **(G)** Examples of thyrocytes from P1 (*top*) and P14 (*bottom*) thyroids. The *left column* shows follicles formed by thyrocytes with various degree of *Tg* TL development; the *right and middle columns* show thyrocytes at a higher magnification with fully or underdeveloped *Tg* TLs. Arrows point at nuclei with underdeveloped loops. Note that in some nuclei only one allele is active. For P1 and P14, image stacks of about 50 and 70 nuclei, respectively, were acquired. Images on B-D and G are projections of 3–5 µm confocal stacks; RNA-FISH and immunostaining signals are *green*; nuclei are counterstained with DAPI (*red*). Scale bars: B, C, E, 2 μm; D, 70 μm; G, follicle overviews on the left, 10 μm, zoomed in nuclei, 5 µm.

It has been recently shown that highly expressed genes expand from their harboring loci and form so called transcription loops (TLs) (Mirny and Solovei, 2021). The expansion of highly expressed genes is attributed to their intrinsic stiffness acquired through decoration of the gene axis with multiple jam-packed RNA polymerases II (RNAPIIs) with attached nascent RNA transcripts (Figure 1A). Therefore, not only the gene length but also the high gene expression level are essential for formation of a microscopically resolvable transcription loop (Leidescher et al., 2022). Exactly this combination of gene properties is extremely rare, especially in mammalian cells. Consequently, TLs had not been described until the recent serendipitous discovery of TLs formed by the unusually upregulated thyroglobulin gene.

The thyroglobulin gene (*Tg*) is expressed exclusively in thyrocytes, which in vertebrates are arranged in follicles that, in turn, united into thyroid glands in most vertebrate groups. The thyroglobulin protein (TG) accumulates in the follicle cavity, representing the major component of the so-called colloid, and serves as a long-storage precursor for synthesis of the thyroid hormones (THs), 3,3′,5,5′-Tetraiodo-L-thyronine (T4) and 3,3′,5′-Triiodo-L-thyronine (T3) (van de Graaf et al., 2001). Importantly, *Tg* is highly upregulated with a transcription level of ca. 23,000 TPM (Transcripts Per Million), exceeding by many fold the expression of other thyrocyte-specific genes, as well as housekeeping genes, e.g., ubiquitinase (6-fold), actin (9-fold), myosin (22-fold) and multiple ribosomal genes (from 10- to 20-fold). Presumably, *Tg* is transcribed in long transcription bursts separated by short infrequent pauses (Figure 1A). In agreement with this, Hi-C analysis of thyroid tissue revealed that *Tg* TL formation perturbs local cohesin-mediated organization by dissolving the *Tg* sub-TAD and reorganizing the larger TAD (see ED Figure 7 in Leidescher et al., 2022).

The presence of two highly extended *Tg* TLs, corresponding to the two active gene alleles in more than 93% of nuclei (Leidescher et al., 2022), indicates that the gene is remarkably upregulated in almost every thyrocyte for a long time. Therefore, we were interested in investigating thyroglobulin gene activity more closely. In particular, we followed *Tg* activation in development and showed that the *Tg* TL is a hallmark of functional thyrocytes. We asked whether the thyroglobulin gene in other vertebrates is similarly upregulated and demonstrated that thyrocytes of all major vertebrate groups exhibit thyroglobulin TLs. We further investigated whether *Tg* is regulated by organismal or cellular physiological conditions and proved that *Tg* transcription and translation are not regulated by the thyroid hormone (TH) status and do not undergo circadian rhythmicity or intron retention, ruling out these possible mechanisms of temporal segregation of the exocrine and endocrine phases.

## Material and methods

### Tissue collection

All mouse studies were executed in accordance with the European Union (EU) directive 2010/63/EU on the protection of animals used for scientific purposes and in compliance with regulations by the respective local Animal Welfare Committees LMU; Committee on Animal Health and Care of the local governmental body of the state of Upper Bavaria; Germany; Animal Welfare Committee of the Landesamt für Natur, Umwelt und Verbraucherschutz Nordrhein-Westfalen (LANUV; Recklinghausen, Germany). CD-1 mice were purchased from Charles River Laboratories, housed in individual cages with free access to food and water on a 12:12 light dark cycle at the Biocenter, Ludwig-Maximilians-University of Munich (LMU). Mice were sacrificed by cervical dislocation after IsoFlo (Isofluran, Abbott) narcosis. *Mct8*-KO mice (Trajkovic et al., 2007), *Trhr1*-KO mice (Rabeler et al., 2004); and *Thrb*-KO mice (Forrest et al., 1996), all on C57BL/6 background, were kept at 22°C in IVC cages in the central animal facility of the University Hospital Essen and had access to normal chow and water *ad libitum*. Adult mutant mice and control littermates were killed by CO_2_ inhalation prior to organ collection.

Human thyroid tissue was sampled from a freshly operated struma according to the ethical consent (ethical approval No. 12-5133-BO to DF) of the Department of Endocrinology, University Hospital Essen, Germany. Thyroids from chicken (*Gallus gallus domesticus*) were collected from white legorn birds. Fertilized eggs of the M11 chicken line were kindly provided by Dr. S. Weigend (Federal Research Institute for Animal Health, Mariensee) and hatched at the Faculty for Veterinary Medicine, Munich. Birds were housed under conventional conditions in aviaries with groups of up to 10 birds and received food and water *ad libitum*. The animals were treated according to the standard protocol approved by The Committee on Animal Health and Care of the local governmental body of the state of Upper Bavaria, Germany. Thyroids from *Xenopus tropicalis* (*Silurana tropicalis*) were collected from four-year-old adult animals. Frogs were purchased at the Centre de Ressources Biologiques de Rennes (CRB) and raised in the PhyMa Laboratory in aquatic housing system (MPAquarien, Rockenhausen, Germany) at 24°C. Generation of transgenic zebrafish (*Danio rerio*) lines expressing fluorophores in thyrocytes, tg (tg:mCherry) and tg (tg:GFP) is described elsewhere (Opitz et al., 2012; Opitz et al., 2013).

In each case, freshly dissected tissues were washed with PBS and then fixed with 4% paraformaldehyde (Carl Roth) solution in PBS for 12–20 h.

### Mouse and human organoids

Generation of functional mouse and human thyroid tissues *in vitro* is described in (Antonica et al., 2012; Romitti et al., 2022). In our experiments we used thyrocyte follicles formed *in vitro* in 3D matrigel culture and follicles formed after grafting differentiated *in vitro* thyrocytes into mouse kidney. All grafting experiments were performed in accordance with local Animal Ethics [Commission d’Ethique du Bien-Être Animal (CEBEA) Faculté de Médecine ULB, Project CMMI-2020-01].

### Primary thyrocyte culture

For cultivating mouse primary thyrocytes *in vitro*, the protocol from Jeker et al., 1999 was adapted. Briefly, thyroid glands of two mice, were minced into pieces using micro scissors under binocular and transferred into 2 mL tube containing 200 U/mL collagenase and 1 U/mL dispase in DMEM/F12/GlutaMax medium. Incubation was performed in a shaking thermo-block at 37°C for 2 h and followed by mechanical disruption using a glass Pasteur pipetting. Then cells were centrifuged at 2,000 rpm (358 g) for 5 min and resuspended in DMEM supplemented with 10% FCS and 3% Pen/Strep. Single thyrocytes and follicles were seeded on coated coverslips (pre-incubated with 1 μg/mL polylysine) and cultured for 30 min, 24 h or 72 h before fixation with 4% formaldehyde.

### Cryosections

After fixation, thyroids were washed with PBS, cryoprotected in a series of sucrose, and embedded in Tissue-Tek O.C.T. compound freezing medium (Sakura). Blocks were stored at −80°C before cutting into 16–20 µm sections using a cryostat (Leica CM3050S). Cryosections were collected on Superfrost Plus slides (Thermo Scientific) and stored at −80°C before use.

### Electron Microscopy

Mouse thyroid glands were fixed with 2% glutaraldehyde in 300 mOsm cacodylate buffer (75 mM cacodylate, 75 mM NaCl, 2 mM MgCl2) for 30 min, postfixed with 1% OsO4 in the same buffer for 1 h at room temperature. After washings in distilled water, samples were incubated in 1% aqueous solution of uranyl acetate (Serva) for 1 h at 4°C, dehydrated in ethanol series and acetone, and embedded in Epon Resin. Thin sections (50–70 nm) were prepared using Reichert Ultracut, stained with Reynolds lead citrate and examined with a transmission electron microscope (JEM 100 SX, JEOL) at 60 kV.

### Gene expression analysis

Dissected thyroids were immediately placed in RNAlater solution and total RNA was isolated using the NucleoSpin RNA Kit (Macherey-Nagel) according to the manufacturer’s instructions. RNA integrity was checked by separating RNA fragments on a 1% agarose gel. Only samples with a 28S:18S rRNA ratio of ∼2:1 and without genomic contamination and RNA degradation were used for downstream applications. 1 μg of total RNA was reverse transcribed according to the manufacturer’s instructions using the High capacity cDNA reverse transcription Kit with random primers (Applied Biosystems) or Maxima H Minus Reverse Transcriptase (Thermo Scientific) with gene specific primers. qPCR was performed in technical and biological triplicates in 10 µL reactions using LightCycler 480 SYBR Green Master Mix (Roche) or Luna Universal qPCR Master Mix (New England Biolabs) according to the manufacturer’s instructions.

### Nanopore sequencing

Freshly dissected thyroids were immediately placed in ice cold TriZol and homogenized using an ultra-turrax dispersing tool. Poly(A)+ transcripts were isolated using magnetic oligoT-beads (Lexogen). The sequencing library was created using the PCR-cDNA Sequencing Kit (PCB111.24, Oxford Nanopore) and sequenced on a PromethION P24 on a R9.4.1 flowcell. The sequencing data were basecalled using Guppy v6.4.6 and mapped to the mouse genome (mm10) using minimap2.

### FISH probes

BAC clones encompassing the thyroglobulin genes and flanking regions of different vertebrate species, as well as *Tshr*, *Slc16a2* and *Tpo* mouse genes, were selected using the UCSC genome browser (see the list of BACs in Table 1) and purchased from BACPAC Resources (Oakland children’s hospital) as agar stabs (https://bacpacresources.org/). BACs were purified via standard alkaline lysis or the NucleoBond Xtra Midi Kit (Macherey-Nagel), followed by amplification with the GenomiPhi Kit (GE Healthcare) according to the manufacturer’s instructions. Amplified BAC DNA was labeled with fluorophores using homemade conjugated fluorophore-dUTPs by nick translation (Cremer et al., 2008). Labeled BAC DNA was ethanol precipitated with 10-fold excess of Cot-1 (1 mg/mL; Invitrogen, 18440-016) and 50-fold excess of salmon sperm DNA (5 μg/μL; Sigma), pellet was dried in a SpeedVak, and dissolved in hybridization mixture containing 50% formamide, 1xSSC and 10% of dextran sulphate. Oligoprobe for the *Tg* mRNA was generated using SABER-FISH protocol and described in detail previously (Leidescher et al., 2022).

**TABLE 1:** BACs used in the study and number of imaged thyrocyte nuclei.

	Latin name	Genomic region	BAC #	Number of imaged nuclei
Mouse	*Mus musculus*	*Tg*, gene body	RP24-229C15	> 3,000
		*Tshr*, gene body	RP23-152C21	32
		*Slc16a2*, gene body	RP23-114F20	29
		*Tpo*, gene body	RP24-172O17	35
Human	*Homo sapience*	*TG*, gene body	RP11-806J1	70
		*TG*, 5′ half	RP11-844M6	
		*TG*, 3′ half	RP11-111C8	
		*TG*, 5′ flank	RP11-599I7	
		*TG*, 3′ flank	RP11-739E11	
Chicken	*Gallus domesticus*	*tg*, gene body	CH261-128F8	38
		*tg*, 5′ flank	CH261-88K23	
		*tg*, 3′ flank	CH261-85H20	
Frog	*Xenopus tropicalis*	*tg*, gene body	CH216-364N2	59
		*tg*, gene body	CH216-26O19	
Zebrafish	*Danio rerio*	*tg*, gene body	CH211-184D12	30
		*tg*, 3′ 3/4 of the gene	CH73-56C22	

### FISH and immunostaining

FISH on cryosections was performed as previously described (Solovei, 2010). For DNA-FISH, sections were treated with 50 μg/mL RNaseA at 37°C for 1 h. For DNA-FISH or FISH detecting DNA and RNA simultaneously, denaturation of both probe and sample DNA was carried out on a hot block at 80°C for 3 min. For only RNA-FISH, RNasing and denaturation steps were omitted. Probes were loaded on sections under small glass chambers and sealed with rubber cement [for detail, see (Solovei, 2010)]. Hybridizations were carried out in a water bath at 37°C for 2 days. After hybridization, rubber cement and chambers were removed, slides with sections were washed with 2xSSC at 37°C, 3 × 30 min, and then with 0.1xSSC at 60°C 1 × 7 min. Hybridized SABER probes were detected by incubating with 1 μM fluorescently labeled detection oligonucleotides in PBS for 1 h at 37°C followed by washing with PBS for 10 min.

The primary antibody for cytoplasmic TG detection (1:50; rabbit anti-TG Abcam, ab 156008) and the secondary donkey anti-rabbit conjugated with Alexa 555 (1:250; Invitrogen, Cat# A31570) were diluted in blocking solution (PBS +2% BSA +0.1% Saponin +0.1% TritonX100) and applied under glass chambers covering sections. Incubations with primary and secondary antibodies were carried overnight at RT, in between and after incubations, sections were washed with PBS +0.05% TritonX100 warmed up to 37°C; 3 × 30 min. In all experiments, nuclei were counterstained with 2 μg/mL DAPI in PBS for 30 min and Vectashield (Vector) was used as an antifade mounting medium.

### Microscopy

Confocal image stacks were acquired using a TCS SP5 confocal microscope (Leica) using a Plan Apo 63/1.4 NA oil immersion objective and the Leica Application Suite Advanced Fluorescence (LAS AF) Software (Leica). Z step size was adjusted to an axial chromatic shift and typically was either 200 nm or 300 nm. XY pixel size varied from 20 to 120 nm. Axial chromatic shift correction, as well as building single grey-scale stacks, RGB-stacks, montages and maximum intensity projections was performed using ImageJ plugin StackGroom (Walter et al., 2006). The plugin is available upon request.

## Results and discussion

### *Tg* is an exceptionally highly expressed gene in thyrocytes

In agreement with its length (180 kb in mouse) and level of transcription (23,000 TPM), the *Tg* gene forms very prominent transcription loops (Figure 1B4). To examine other long thyroid-specific genes, we selected thyroid peroxidase (*Tpo*) with a length of 78 kb, the TH transporting monocarboxylate transporter MCT8 (*Slc16a2*) that is 125 kb long and the thyroid stimulating hormone receptor (*Tshr*) with a length of 139 kb. The RNA signals of *Slc16a2* and *Tshr*, which have comparable length and ca. 100-500 times lower expression levels than *Tg*, are small and only slightly elongated. On the contrary, the much shorter *Tpo* gene, which is about 80 kb long but expressed at ca. 550 TPM, noticeably expands and forms short but distinctive transcription loops (Figure 1B). This finding confirms that formation of microscopically resolvable transcription loops is dependent not only on the gene length, but also on the intensity of transcription (Leidescher et al., 2022).

Remarkably, every single thyrocyte in mouse thyroid manifests *Tg* transcription, and the majority of cells possess two TLs, with only 6.7% of thyrocytes exhibiting monoallelic expression (Leidescher et al., 2022). The high transcription of the *Tg* gene is consistent with high production of the thyroglobulin protein (TG). The *Tg* transcripts comprise 2.5% of the entire mRNA pool in mouse thyroid, similarly to 2.6% shown for human thyrocytes (Pauws et al., 2000). In accordance, the probe for *Tg* mRNA highlights not only *Tg* TLs and intranuclear mRNA, but also the whole thyrocyte cytoplasm (Figure 1C). In agreement with this, the cytoplasm is brightly stained with an antibody against TG (Figure 1D), and exhibits dilated cisternae of the Golgi apparatus, as well as a remarkable hypertrophy of the rough endoplasmic reticulum, which occupies most of the thyrocyte cytoplasm (Figure 1E). The above observations corroborate the statement that the *Tg* gene is abnormally upregulated and thyrocytes produce immense amounts of *Tg* mRNAs and TG protein.

### Thyrocytes exhibit *Tg* TLs from the onset of their differentiation

In mouse, the onset of folliculogenesis starts at E15.5 and the thyroid gland forms before birth (De Felice and Di Lauro, 2011). We aimed at estimating the *Tg* gene expression in early postnatal development and performed qPCR on thyroid glands dissected at stages P1 and P14. We showed that *Tg* expression increases during development, reaching a 3.3-fold rise in the adult mice compared to P1 pups (Figure 1F). RNA-FISH revealed that although *Tg* TLs in most P1 and P14 thyrocytes are fully developed, ca. 20% and 12% of the cells, respectively, possessed small, undeveloped loops and often only one active allele (Figure 1G, arrows). Apparently, these cells represent freshly differentiated or still differentiating thyrocytes. Although mitotic cells in the follicle epithelium are relatively infrequent, in both developmental stages, we observed several thyrocytes entering or exiting the cell cycle. In particular, we noticed that *Tg* TLs are manifested when most of the nuclear chromatin is condensed: they appear in early G1, remain visible in thyrocyte nuclei until early-to-mid prophase and are withdrawn only in the very late prophase (Supplementary Figure S1). Thus, we conclude, that the *Tg* gene is expressed at a high level from the moment cells acquire thyrocyte identity.

### The *Tg* gene is highly expressed and forms transcription loops in all vertebrates

Initially, the thyroglobulin TLs have been studied in mouse thyrocytes (Leidescher et al., 2022). However, THs are essential regulatory elements in the development and metabolism of all animals, and the mechanisms of TH generation are evolutionary conserved (Di Jeso and Arvan, 2016; Holzer et al., 2016). Therefore, we anticipated that in other mammals and other vertebrate classes, expression of thyroglobulin gene orthologues is also highly upregulated.

**FIGURE 2:**
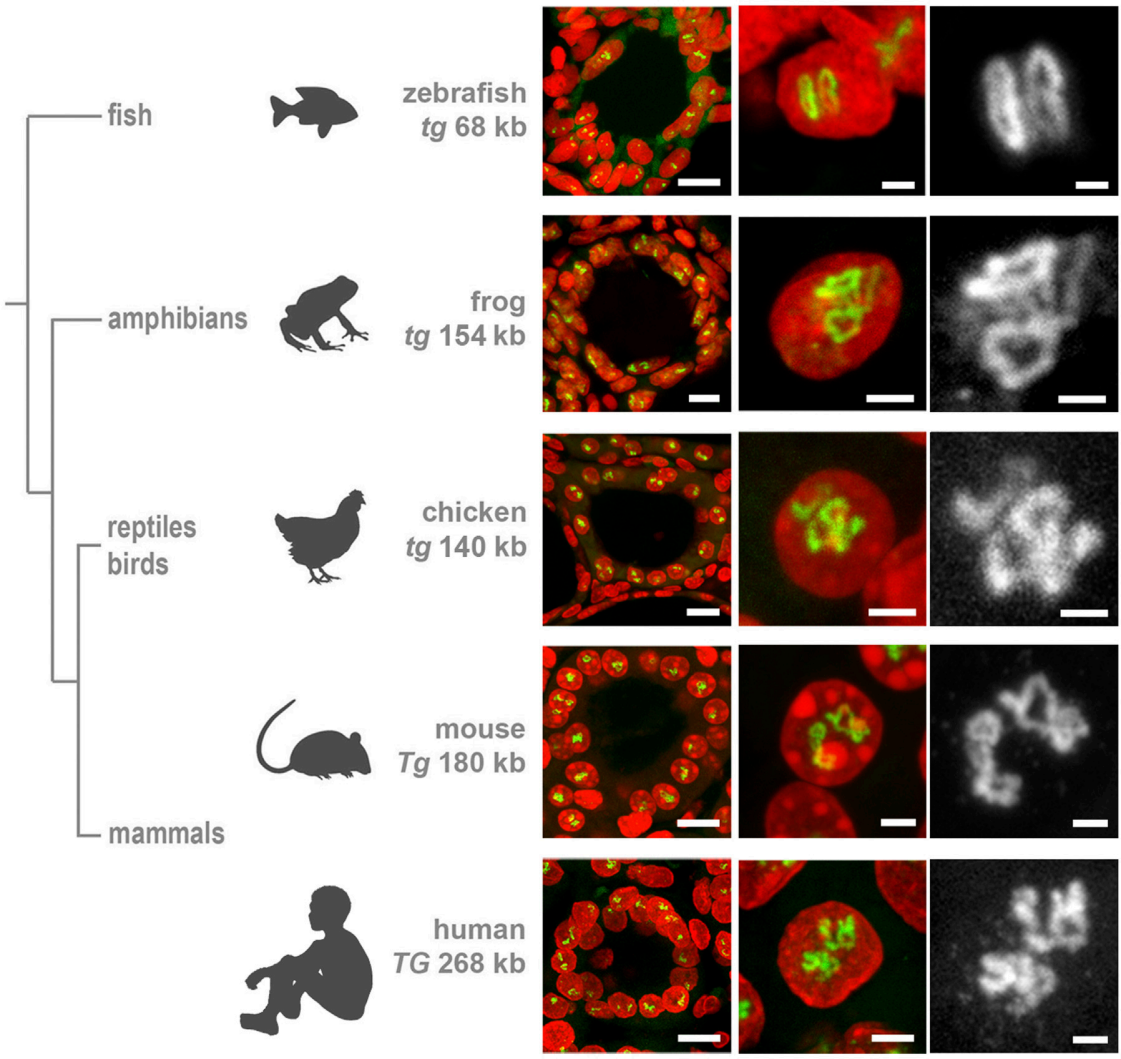
Thyroglobulin TLs are a peculiarity of thyrocytes from all major vertebrate groups. RNA-FISH with species-specific probes hybridizing to nascent RNA transcripts of thyroglobulin genes reveals TLs in all studied species. *The left column* shows thyroid follicles; *the mid column* shows representative thyrocyte nuclei at a higher magnification, *the right column* shows close-ups of *Tg* TLs of the middle column as greyscale images. Note that even the shortest among vertebrates, zebrafish *tg* with a length of only 68 kb, forms TLs resolvable by light microscopy. For the number of analyzed nuclei for each species, see Table 1. RNA-FISH signals (*green*), DAPI (*red*); images are projections of confocal 3–6 µm stacks. Scale bars: left column, 10 μm; mid column, 2 μm; right column, 1 µm.

First, we demonstrated that human *TG* (length of 280 kb) exhibits TLs in thyrocytes of a freshly operated human thyroid (Figure 2). Next, we detected the thyroglobulin gene (*tg*) in the chicken *Gallus domesticus* (length of ca.140 kb) and the frog *Xenopus tropicals* (length of ca.154 kb), verifying that the gene forms TLs in these species as well (Figure 2). Finally, we aimed at visualization of the thyroglobulin gene in fish species, where thyrocytes are assembled into single follicles, which are not gathered into a gland, remain separated and are scattered along the esophagus. Therefore, to overcome the difficulties of finding follicles, we made use of the transgenic zebrafish *Danio rerio* lines generated previously, in which thyrocytes express fluorophores under the *tg* promoter, tg (*tg*:mCherry) or tg (*tg*:GFP) (Opitz et al., 2012; Opitz et al., 2013). Using cryosections from these transgenic lines, we demonstrated that, despite the relatively small length of the *D*.*rerio tg* (68 kb), it forms microscopically resolvable TLs strongly expanding throughout the nucleus (Figure 2).

**FIGURE 3:**
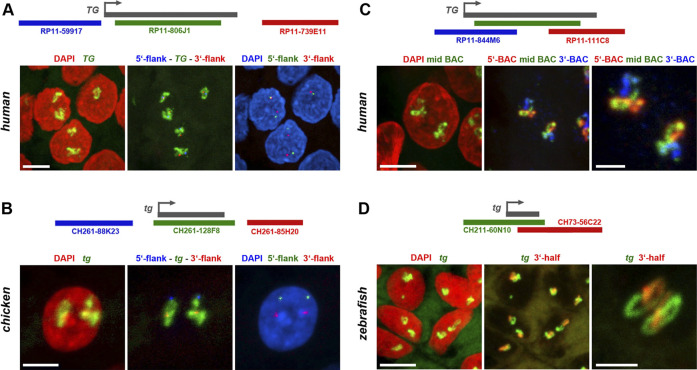
Thyroglobulin TLs in other vertebrates exhibit the key features of TLs. **(A,B)** As exemplified by simultaneous RNA- and DNA-FISH for human **(A)** and chicken **(B)** thyroglobulin, their TLs are open loops with visibly separated flanks. Images on the *left* show thyrocyte nuclei (*red*) with entire TLs (*green*). *Mid* images show the same TLs in *green* and the two flanks in *blue* (5′) and *red* (3′). Images on the *right*, for better demonstration of flank separation, show the same nuclei (blue) and the two flanks in *green* (5′) and *red* (3′). **(C,D)** As exemplified by RNA-FISH for human **(C)** and zebrafish **(D)** thyroglobulin genes, genomic probes highlighting introns, label TLs sequentially as a result of a co-transcriptional splicing. *Left* images show thyrocyte nuclei (*red*) with entire TLs (*green*). *Mid* images show the same TLs after hybridization with mid (*green*), 5’ (*blue*) and 3’ (*red*) BACs **(C)**. For zebrafish *tg*, only two BACs were used, one covering the whole gene (*green*) and one—its 3′ half (*red*) **(D)**. Images on the *right* show TLs at a higher magnification with the same colour code. Between 20 and 40 nuclei were acquired for each of the experiments. Schematics of genes and used BACs are shown above every panel. Images are projections of confocal stacks through 6 µm **(A,C)**, 3.5 µm **(B)** and 2.5 µm **(D)**. Scale bars: A-D, 5 μm; right panels on C and B, 2 µm.

Importantly, all the characteristic TL features, shown previously only for mouse TLs, are utterly manifested in other species as well. Firstly, we observed separation of TL flanks (Figures 3A, B), which has been previously described for other highly expressed long genes and is presumably explained by increased gene stiffness (Leidescher et al., 2022). Secondly, it has been demonstrated that probes highlighting introns over long gene stretches label TLs sequentially as a result of co-transcriptional splicing (Leidescher et al., 2022). This phenomenon is now also confirmed for human and fish thyroglobulin TLs by using sequential BAC probes hybridizing mostly to introns of nascent RNA transcripts decorating the loops (Figures 3C, D).

These data show a high upregulation of the thyroglobulin gene in the major vertebrate classes confirming the key role of TG in hormone production. This conclusion is reinforced by the recent work demonstrating a highly conserved structure of TG protein among vertebrates with all domains conserved from basal vertebrates to mammals (Di Jeso and Arvan, 2016; Holzer et al., 2016). Moreover, the authors of this paper conclude that since no orthologues of thyroglobulin gene have been found in invertebrates, TG protein and the mechanism for TH formation are vertebrate inventions.

### *Tg* TLs are a robust mark of thyrocytes differentiated *in vitro*

Sabine Costagliola’s research group described the generation of a functional thyroid tissue *in vitro* in 2012 (Antonica et al., 2012). The authors demonstrated that transient overexpression of the transcription factors Nkx2-1 and Pax8 in mouse embryonic stem cells, followed by 3D culturing, allows the generation of follicles similar in morphology and gene expression to the thyroid follicles *in vivo*. 10 years later, the same group using similar strategy succeeded in generating and growing human thyroid organoids (Romitti et al., 2022). To confirm the functionality of mouse and human follicles generated *in vitro*, cultured follicles of both species were grafted into kidneys of athyroid mice resulting in successful rescue of hormone production after 4–5 weeks.

**FIGURE 4:**
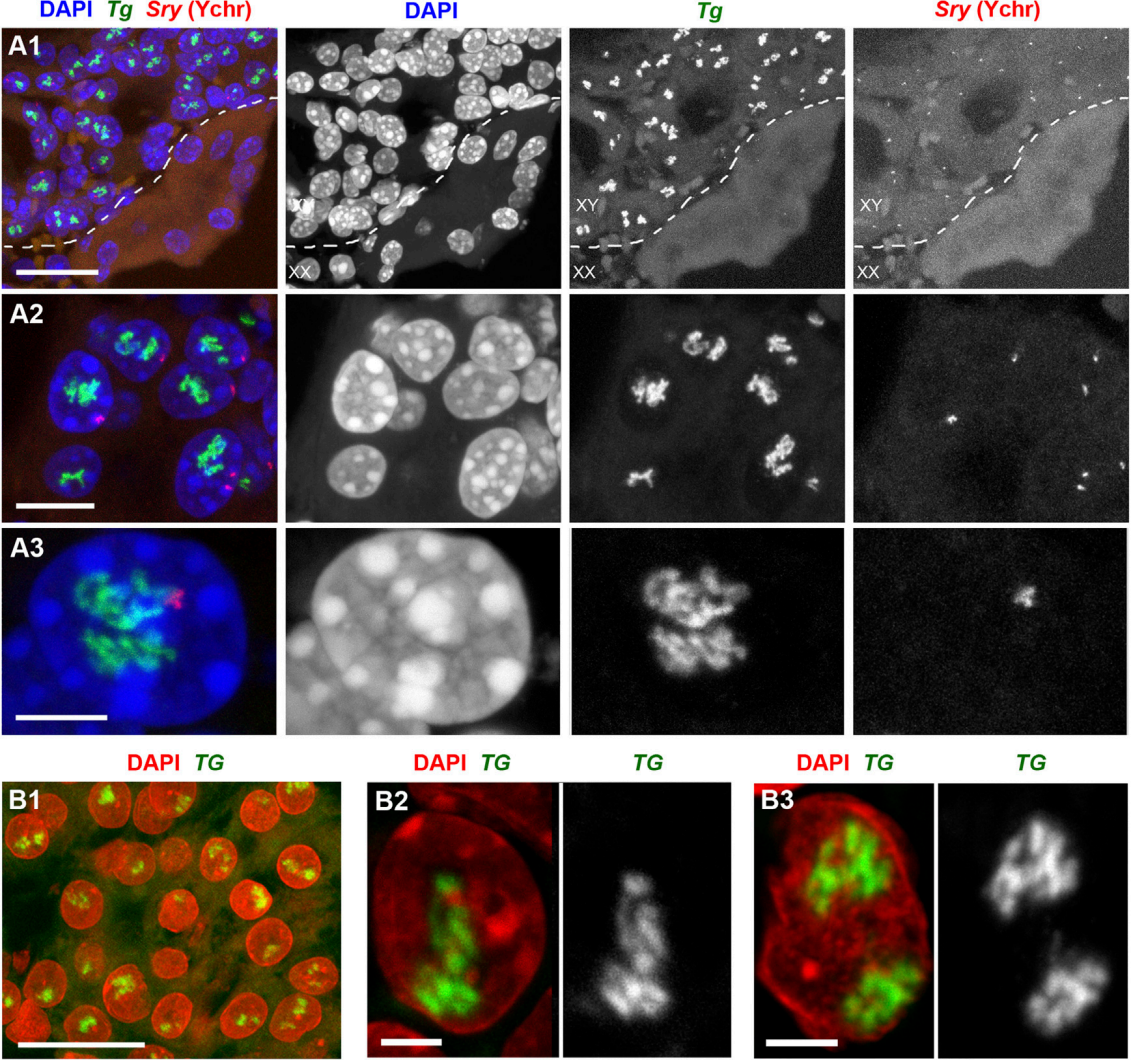
Mouse **(A)** and human **(B)** thyroid organoids grafted into mouse kidney. Cells of grafted follicles, generated from mouse male ESCs, can be distinguished from female mouse host cells by DNA-FISH with a Y chromosome-specific probe (*red*). The dotted line in **(A1)** separates host kidney cells from thyrocytes of follicles. Note the high extension of *Tg* and *TG* TLs in comparison to TLs formed by these genes in cultured follicles (compare to Supplementary Figure S2). 30 and 32 stacks were acquired from mouse and human grafted thyroid organoids, respectively. Thyroglobulin TLs are *green*; DAPI is *blue* in **(A)** and *red* in **(B)**. Images are projections of confocal 4 µm stacks **(A1,B1)** and 2.5 µm **(A2,A3,B2,B3)**. Scale bars: **(A1,B)** 25 μm, **(A2)** 10 μm, **(A3)** 5 μm, **(B2,B3)** 2.5 µm.

Not surprisingly, RNA-FISH revealed *Tg* TLs in mouse and human follicles cultured *in vitro*, although the loop size was noticeably smaller in comparison to thyrocytes in glands (Supplementary Figure S2). To visualize mouse *Tg* TLs in the grafted thyrocytes, we used RNA- and DNA-FISH simultaneously—to identify injected male mouse cells in the female host tissue by a probe for the Y chromosome (DNA-FISH) and to detect *Tg* loops (RNA-FISH). Accumulations of small follicles were clearly distinguished from the host kidney tissue by Y chromosome DNA-FISH signals (Figure 4A), and predictably, only cells with this marker exhibited *Tg* TLs, which were much more extended compared to cultured follicles or even thyrocytes within the thyroid gland (Figures 4A2, 4A3).

Differentiation between host mouse cells and human cells grafted into mouse kidney is an easy task, because in difference to human, mouse nuclei possess multiple chromocenters formed by subcentromeric major satellite repeat (Vissel and Choo, 1989; Erdel et al., 2020) brightly stained with DAPI (Supplementary Figure S3A). In addition, transgenic differentiated thyrocytes express NKX2-GFP and thus could be distinguished from non-thyrocytes (Supplementary Figure S3B). Similarly to mouse, human grafted thyrocytes form follicles (Figure 4B1) and the *TG* genes become strongly expanded (Figures 4B2, 4B). What is more, expressed *TG* modifies the harboring chromosomal locus by pushing its flanks away from each other (Supplementary Figure S3C) in a manner described for other resolvable TLs (Leidescher et al., 2022). Interestingly, using probes for the gene body and flanks, we noticed that several thyrocytes had four instead of two *TG* TLs (Supplementary Figure S3C), indicating that either during culturing *in vitro* or after grafting, some human thyrocytes became tetraploid. The *TG* TLs exhibit another typical TL feature, the co-transcriptional splicing: the three consecutive overlapping genomic probes sequentially label the nascent RNAs decorating the gene because the introns are sequentially spliced out (Supplementary Figure S3D). Thus, the work with thyrocytes differentiated from ESCs confirms that thyroglobulin TL formation is an invariable mark of differentiated thyrocytes.

### Thyrocytes are functional only in follicles

The noticeably stronger extension of both *Tg* and *TG* TLs in grafts in comparison to thyrocytes *in vitro* indicates a higher upregulation of the gene within an organism. Culturing of single thyrocytes isolated from thyroid confirms the importance of the follicle structure for thyrocyte functional activity. Following the protocol by (Jeker et al., 1999) for thyroid disintegration and culturing (see Methods), we generated primary transient cultures of mouse thyrocytes, consisting mostly of single thyrocytes and remnants of follicles. Immediately after disintegration, cells were attached to coverslips, fixed and hybridized with a *Tg* genomic probe. RNA-FISH showed that thyrocytes exhibit *Tg* TLs for at least an hour after follicle disintegration (Supplementary Figures S4A, S4B). After 24 h of incubation, however, single thyrocytes became flatter and lost *Tg* TLs (Supplementary Figure S4C), although strongly reduced TLs were still present in some cells within the remaining flattened follicles. At this stage of the culture, no dividing cells were observed. After 72 h of incubation, all thyrocytes migrated out of follicles, became very flat, exhibited proliferative activity (with a mitotic index of 2.7%) and formed a monolayer, in which not a single cell exhibited *Tg* TLs (Supplementary Figure S4D). Apparently, thyrocytes lost their identity, possibly de-differentiated and entered the cell cycle as reported earlier for thyrocyte cultures established from other vertebrates (Kimura et al., 2001). These data indicate that results of various analyses conducted on cultured thyrocytes, as well as on other differentiated cells transferred to *in vitro* conditions, must be treated cautiously.

### *Tg* expression is independent of thyroidal TH status

**FIGURE 5:**
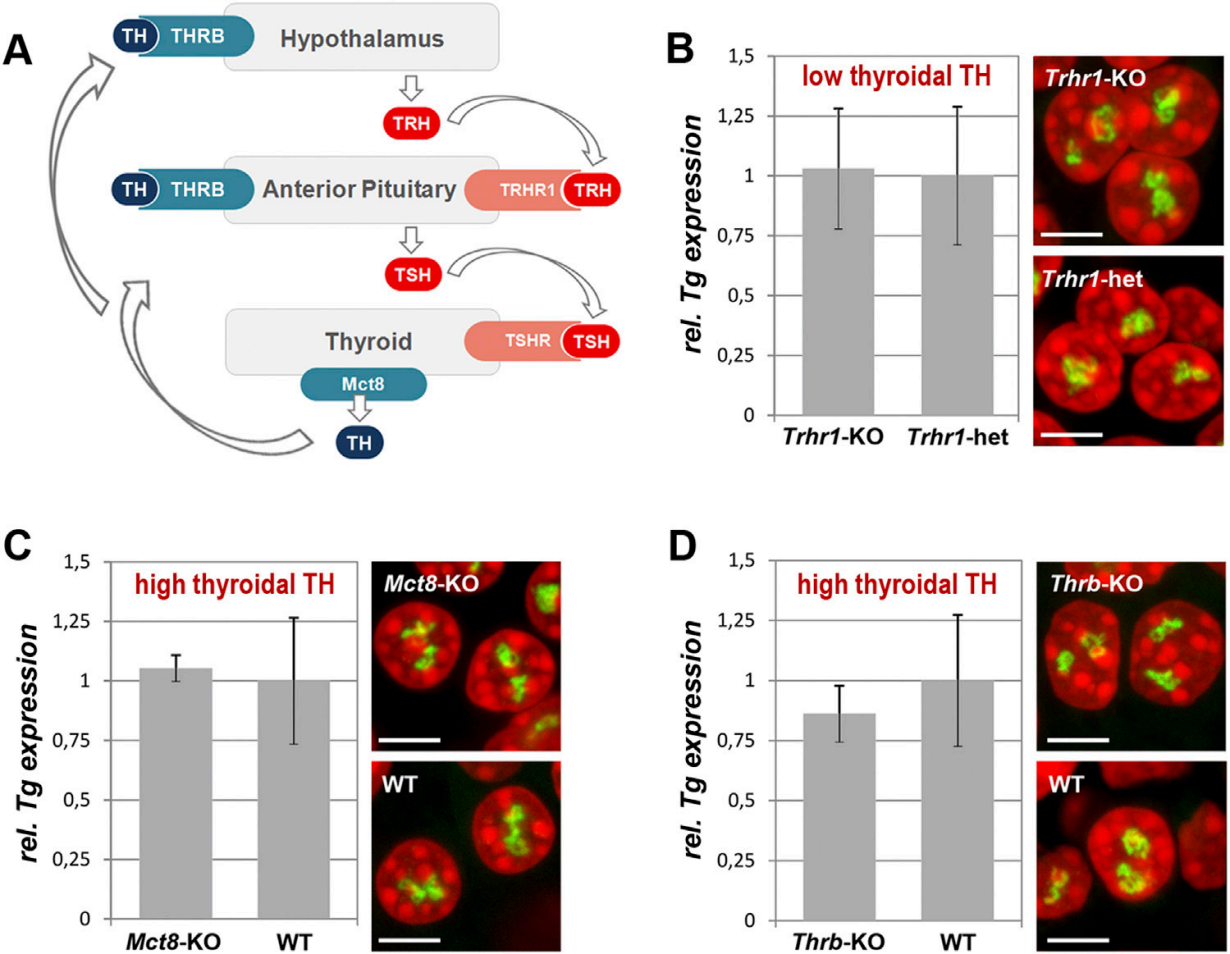
Thyroidal TH status does not influence *Tg* expression. **(A)** Simplified schematics of the hypothalamus-pituitary-thyroid axis. *TH*, thyroid hormones; *THRB*, TH receptor; *TRH*, hypothalamic thyrotropin-releasing hormone; *TRHR1*, thyrotropin-releasing hormone receptor; *TSH*, thyroid stimulating hormone; *TSHR*, thyroid stimulating hormone receptor. **(B–D)** Results of qPCR detecting levels of *Tg* expression (*left panels*) and RNA-FISH detecting *Tg* TLs (*right panels*) at a decreased **(B)** and increased **(C–D)** TH production in comparison to control mice. *Tg* transcript levels were normalized to the transcription level of the thyroid specific transcription factor Pax8 (see explanation in Figure 1F legend). Three biological replicates were analyzed per gene. All observed changes were not significant (*p* > 0.05) as determined by Wilcoxon rank sum test. For each of the conditions, two replicates were used for microscopy; between 20 and 30 confocal stacks with one or two nuclei were acquired. For more examples of nuclei, see Supplementary Figure S5. Bars in graphs are SDM. *Tg* TLs, *green*; DAPI, *red*; images are projections of 2–3 µm confocal stacks; scale bars: 5 µm.

TH production is tightly regulated by the activity of the hypothalamus-pituitary-thyroid axis and controlled by negative feedback loops involving the TH receptor THRB (Ortiga-Carvalho et al., 2016). Hypothalamic thyrotropin-releasing hormone (TRH) activates its pituitary TRHR1 receptor and stimulates the thyroid by stimulating hormone (TSH) release, which in turn acts on TSHR of thyrocytes and stimulates the production and secretion of THs (Figure 5A). However, data on the regulation of the *Tg* gene activity are controversial. Earlier works showed that continuous presence of TSH is required to maintain TG production (Van Heuverswyn et al., 1984; Kim and Arvan, 1993). More recent work, however, showed that TSH deprivation or lack of functional TSHR due to early development does not affect the *Tg* expression but greatly reduces the expression of thyroperoxidase and the sodium/iodide symporter (Postiglione et al., 2002).

To test whether *Tg* expression is regulated by the thyroidal TH status, we sampled thyroids from mice with increased or decreased thyroidal TH production. For an increased thyroidal production we investigated *Mct8*-KO mice that exhibit a highly increased thyroidal TH content and reduced thyroidal T4 secretion (Trajkovic et al., 2007; Di Cosmo et al., 2010; Trajkovic-Arsic et al., 2010) (Figures 5A, C) as well as *Thrb*-KO mice that show highly elevated TSH and TH levels (Forrest et al., 1996) (Figures 5A, D). We also examined *Trhr1*-KO mice that display central hypothyroidism with decreased thyroidal and serum TH concentrations (Rabeler et al., 2004; Groba et al., 2013) (Figures 5A, B). For each condition, three thyroids were fixed for RNA-FISH and other three used for qPCR.

We anticipated to observe a drop of *Tg* transcription level and consequently a decrease in *Tg* TL size under “hyper” conditions, whereas “hypo” condition with decreased thyroidal TH concentration might show elevated *Tg* transcription level and thus increased *Tg* TL size. In all three conditions, however, *Tg* mRNA level did not differ from that of control samples and thyrocytes exhibited *Tg* TLs of the size and morphology similar to those in control animals (Figures 5B–D). Based on these results, we concluded that the intrathyroidal TH status does not influence *Tg* expression and that the gene is perpetually upregulated regardless of the activity of the hypothalamus-pituitary-thyroid axis.

### *Tg* is upregulated during both the exocrine and endocrine activities of thyrocytes

**FIGURE 6:**
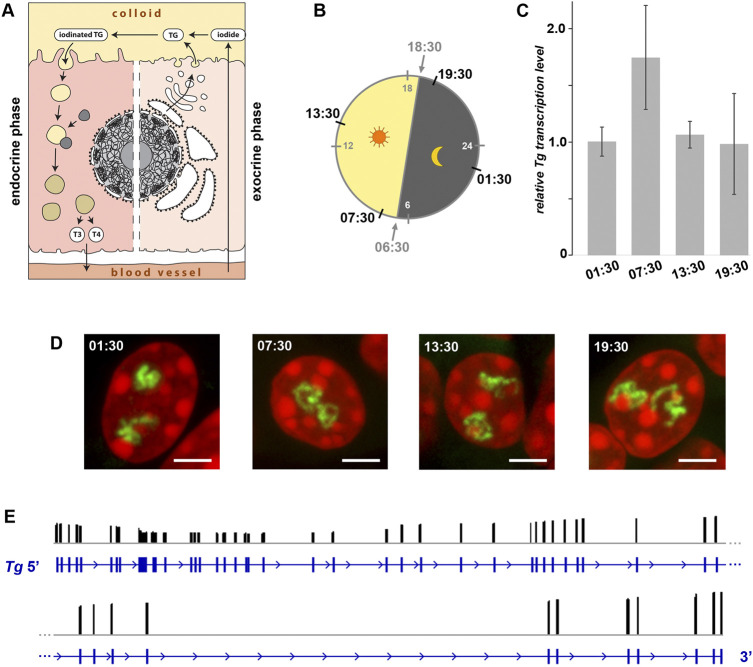
Expression of the *Tg* gene is not regulated by circadian activity and intron retention. **(A)** Schematics of a thyrocyte with two phases depicted - exocrine (*right*) and endocrine (*left*). **(B)** Diagram showing light: dark schedule (grey numbers) and time points (black numbers) of thyroid sampling for the circadian activity experiment. **(C)** Results of qPCR detecting levels of *Tg* expression indicate that there is no significant difference between the four time points. Bars are SDM. Expression levels were normalized to the 01:30 time point. Three biological replicates were analyzed per time point. The observed changes were not significant (*p* > 0.05) as determined by one-way ANOVA with Tukey’s multiple comparisons *post hoc*. **(D)** Typical examples of thyrocyte nuclei after RNA-FISH detecting *Tg* TLs at four time points. *Tg* TLs, *green*; DAPI, *red*; images are projections of 3 µm confocal stacks; scale bars: 5 µm. For each of the three replicates at each time-point, between 40 and 50 confocal stacks were acquired. For more examples of nuclei, see Supplementary Figure S6. **(E)** Results of Nano-pore sequencing of poly(A) fraction for the *Tg* gene. RNA-seq read coverage (*black columns*) across the gene locus (*blue*). The read coverage is ranging between 0 and 3,146; only exon gene regions coincide with reads. For better presentation, the *Tg* gene is divided in two-halves that are shown one under the other.

The thyrocytes function as both exocrine and endocrine glands. On the apical side, a thyrocyte secretes proteins (e.g., TG, TPO) into the follicle cavity filled with colloid, and on the basolateral site it releases TH into the circulation (Figure 6A). Surprisingly, the question whether these two phases of activity happen simultaneously or during separate time-windows remains open. One possibility to separate the phases would be a regulated oscillation in synthesis and excretion of TG, which is the major component of the follicle colloid, due to a potential *Tg* gene circadian rhythmicity. Indeed, human TSH exhibits a clear circadian rhythm with a peak between 2 and 4 a.m. (Russell et al., 2008). This fact suggests that the *Tg* gene might also be subject to circadian activity, separating in this way the two thyrocyte physiological phases.

To check the possible oscillation of the *Tg* gene, we assessed the abundance of *Tg* RNAs and the presence of *Tg* transcription loops in thyroid glands from healthy mice round-the-clock. The mice were entrained to a 12 h: 12 h light: dark schedule and sacrificed at four time points according to one of the standard schemes for circadian activity testing. The time points included 1 hour after the light switched on (07:30), 1 hour after the light switched off (19:30), and two time points in the middle (13:30 and 01:30) (Figure 6B). Thyroids of three animals were sampled for each time point, with one lobe of each thyroid used for qPCR to estimate *Tg* mRNA level and the other lobe used for microscopy.

In the first part of our analysis, using qPCR, we found that levels of Tg mRNA at the four time points were not significantly different (Figure 6C), indicating continuing high transcription and lack of acute waves of mRNA decay (Garneau et al., 2007). However, since the mRNA was extracted from entire cells (more accurately, from entire thyroid glands), our analysis included mixture of cytoplasmic and nucleoplasmic RNA fractions. Therefore, since Tg mRNA is super-abundant in the cytoplasm and apparently less so in nuclei, from such analysis we could not conclude that Tg transcription is not altered between the time points.

To clarify this question, in the second part of our analysis, we performed RNA-FISH highlighting nascent RNA transcripts and found that thyrocytes exhibit well developed Tg TLs at all four time points (Figure 6D and Supplementary Figure 6). Presence of TLs unlikely can be explained by “frozen” or “slowed down” transcription, because speed of RNAPII machinery is known to be pretty stable (3.8 kb/min(^−1^); Singh and Padgett, 2009), and therefore, microscopy control demonstrates that Tg transcription is indeed going on round the clock. According to the above data, we conclude that the Tg gene is not rhythmically expressed and remains highly upregulated throughout 24 h.

The presence of extended *Tg* TLs in thyrocytes round-the-clock still leaves an opportunity for the regulation of gene expression by accumulation of transcripts in the nucleoplasm followed by their concurrent export into the cytoplasm. Such mechanism of fine gene expression tuning is known as the intron retention phenomenon described for various cell types (Braunschweig et al., 2014). Intron retention allows an acute release of mRNA into the cytoplasm by synchronous excision of retained introns in response to a stimulus (Mauger et al., 2016). Thus, despite the perpetual transcription of *Tg*, intron retention might be a mechanism for *Tg* mRNA accumulation within nuclei during the endocrine phase and their release during the exocrine phase. To test this hypothesis, we isolated RNA from three mouse thyroid glands and performed Nanopore sequencing. As shown in Figure 6E, we have not detected intron retention in the poly(A) fraction of RNA: all sequenced reads mapped exclusively to the gene exons, which indicates that introns of all *Tg* mRNAs are excised. Therefore, we can rule out intron retention as a mechanism for restricting the TG production window.

## Conclusion

Collectively, our data suggest that both thyroglobulin alleles are perpetually upregulated in thyrocytes of the major vertebrate groups with a seemingly everlasting expression during the entire cell life. On one hand, it underscores the physiological importance of TH for proper organismal development and function. On the other hand, in view of the large volumes of TG stored within follicles, such high and enduring expression of the *Tg* gene, at any condition, at any age and round-the-clock seems counterintuitive and remains enigmatic.

Conceivably, it might reflect an inefficient way of hormone production evolved during vertebrate evolution. Indeed, only a small proportion of the 66 tyrosine residues of the thyroglobulin molecule becomes iodinated and only three or four TH molecules result from cleavage of one thyroglobulin molecule during thyrocyte endocrine activity (van de Graaf et al., 2001; Di Jeso and Arvan, 2016). Therefore, such wasteful thyroglobulin production might be the way to correct this unintelligible nature design. Another not mutually exclusive explanation of the phenomenon is that massive TG production is needed for storage of the rare trace element iodine (Crockford, 2009). One can speculate that binding to a large protein is a safe way of building an iodine reservoir within an organism.

Taking in account the very low turnover of thyrocytes (Dumont et al., 1992), we deduce that the thyroglobulin gene is perpetually active, e.g., for months in mouse and for years in human (Coclet et al., 1989). In this respect, the phenomenon of the thyroglobulin gene represents an attractive model to study transcription regulation, in particular, molecular mechanisms of high upregulation maintenance, chromatin dynamics, and kinetics of splicing.

## Data Availability

The datasets presented in this study can be found in online repositories. The names of the repository/repositories and accession number(s) can be found below: https://www.ncbi.nlm.nih.gov/geo/, GSE233457.

## References

[B1] Antonica F. Kasprzyk D. F. Opitz R. Iacovino M. Liao X. H. Dumitrescu A. M. (2012). Generation of functional thyroid from embryonic stem cells. Nature 491, 66–71. 10.1038/nature11525 23051751 PMC3687105

[B2] Braunschweig U. Barbosa-Morais N. L. Pan Q. Nachman E. N. Alipanahi B. Gonatopoulos-Pournatzis T. (2014). Widespread intron retention in mammals functionally tunes transcriptomes. Genome Res. 24, 1774–1786. 10.1101/gr.177790.114 25258385 PMC4216919

[B3] Coclet J. Foureau F. Ketelbant P. Galand P. Dumont J. E. (1989). Cell population kinetics in dog and human adult thyroid. Clin. Endocrinol. (Oxf) 31, 655–665. 10.1111/j.1365-2265.1989.tb01290.x 2627756

[B4] Cremer M. Grasser F. Lanctot C. Muller S. Neusser M. Zinner R. (2008). Multicolor 3D fluorescence *in situ* hybridization for imaging interphase chromosomes. Methods Mol. Biol. 463, 205–239. 10.1007/978-1-59745-406-3_15 18951171

[B5] Crockford S. J. (2009). Evolutionary roots of iodine and thyroid hormones in cell-cell signaling. Integr. Comp. Biol. 49, 155–166. 10.1093/icb/icp053 21669854

[B6] De Felice M. Di Lauro R. (2011). Minireview: intrinsic and extrinsic factors in thyroid gland development: an update. Endocrinology 152, 2948–2956. 10.1210/en.2011-0204 21693675

[B7] Di Cosmo C. Liao X. H. Dumitrescu A. M. Philp N. J. Weiss R. E. Refetoff S. (2010). Mice deficient in MCT8 reveal a mechanism regulating thyroid hormone secretion. J. Clin. Invest 120, 3377–3388. 10.1172/JCI42113 20679730 PMC2929715

[B8] Di Jeso B. Arvan P. (2016). Thyroglobulin from molecular and cellular Biology to clinical Endocrinology. Endocr. Rev. 37, 2–36. 10.1210/er.2015-1090 26595189 PMC4740344

[B9] Dumont J. E. Lamy F. Roger P. Maenhaut C. (1992). Physiological and pathological regulation of thyroid cell proliferation and differentiation by thyrotropin and other factors. Physiol. Rev. 72, 667–697. 10.1152/physrev.1992.72.3.667 1320763

[B10] Erdel F. Rademacher A. Vlijm R. Tunnermann J. Frank L. Weinmann R. (2020). Mouse heterochromatin adopts digital compaction states without showing hallmarks of HP1-driven liquid-liquid phase separation. Mol. Cell 78, 236–249. 10.1016/j.molcel.2020.02.005 32101700 PMC7163299

[B11] Forrest D. Hanebuth E. Smeyne R. J. Everds N. Stewart C. L. Wehner J. M. (1996). Recessive resistance to thyroid hormone in mice lacking thyroid hormone receptor beta: evidence for tissue-specific modulation of receptor function. EMBO J. 15, 3006–3015. 10.1002/j.1460-2075.1996.tb00664.x 8670802 PMC450242

[B12] Garneau N. L. Wilusz J. Wilusz C. J. (2007). The highways and byways of mRNA decay. Nat. Rev. Mol. Cell Biol. 8, 113–126. 10.1038/nrm2104 17245413

[B13] Groba C. Mayerl S. Van Mullem A. A. Visser T. J. Darras V. M. Habenicht A. J. (2013). Hypothyroidism compromises hypothalamic leptin signaling in mice. Mol. Endocrinol. 27, 586–597. 10.1210/me.2012-1311 23518925 PMC5416808

[B14] Holzer G. Morishita Y. Fini J. B. Lorin T. Gillet B. Hughes S. (2016). Thyroglobulin represents a novel molecular architecture of vertebrates. J. Biol. Chem. 291, 16553–16566. 10.1074/jbc.M116.719047 27311711 PMC4974371

[B15] Jeker L. T. Hejazi M. Burek C. L. Rose N. R. Caturegli P. (1999). Mouse thyroid primary culture. Biochem. Biophys. Res. Commun. 257, 511–515. 10.1006/bbrc.1999.0468 10198242

[B16] Kim P. S. Arvan P. (1993). Hormonal regulation of thyroglobulin export from the endoplasmic reticulum of cultured thyrocytes. J. Biol. Chem. 268, 4873–4879. 10.1016/s0021-9258(18)53477-3 8095263

[B17] Kimura T. Van Keymeulen A. Golstein J. Fusco A. Dumont J. E. Roger P. P. (2001). Regulation of thyroid cell proliferation by TSH and other factors: A critical evaluation of *in vitro* models. Endocr. Rev. 22, 631–656. 10.1210/edrv.22.5.0444 11588145

[B18] Leidescher S. Ribisel J. Ullrich S. Feodorova Y. Hildebrand E. Galitsyna A. (2022). Spatial organization of transcribed eukaryotic genes. Nat. Cell Biol. 24, 327–339. 10.1038/s41556-022-00847-6 35177821 PMC9380065

[B19] Mauger O. Lemoine F. Scheiffele P. (2016). Targeted intron retention and excision for rapid gene regulation in response to neuronal activity. Neuron 92, 1266–1278. 10.1016/j.neuron.2016.11.032 28009274

[B20] Mirny L. A. Solovei I. (2021). Keeping chromatin in the loop(s). Nat. Rev. Mol. Cell Biol. 22, 439–440. 10.1038/s41580-021-00337-x 33504981

[B21] Opitz R. Antonica F. Costagliola S. (2013). New model systems to illuminate thyroid organogenesis. Part I: an update on the zebrafish toolbox. Eur. Thyroid. J. 2, 229–242. 10.1159/000357079 24783054 PMC3923603

[B22] Opitz R. Maquet E. Huisken J. Antonica F. Trubiroha A. Pottier G. (2012). Transgenic zebrafish illuminate the dynamics of thyroid morphogenesis and its relationship to cardiovascular development. Dev. Biol. 372, 203–216. 10.1016/j.ydbio.2012.09.011 23022354

[B23] Ortiga-Carvalho T. M. Chiamolera M. I. Pazos-Moura C. C. Wondisford F. E. (2016). Hypothalamus-pituitary-thyroid Axis. Compr. Physiol. 6, 1387–1428. 10.1002/cphy.c150027 27347897

[B24] Pauws E. Moreno J. C. Tijssen M. Baas F. De Vijlder J. J. Ris-Stalpers C. (2000). Serial analysis of gene expression as a tool to assess the human thyroid expression profile and to identify novel thyroidal genes. J. Clin. Endocrinol. Metab. 85, 1923–1927. 10.1210/jcem.85.5.6532 10843176

[B25] Postiglione M. P. Parlato R. Rodriguez-Mallon A. Rosica A. Mithbaokar P. Maresca M. (2002). Role of the thyroid-stimulating hormone receptor signaling in development and differentiation of the thyroid gland. Proc. Natl. Acad. Sci. U. S. A. 99, 15462–15467. 10.1073/pnas.242328999 12432093 PMC137739

[B26] Rabeler R. Mittag J. Geffers L. Ruther U. Leitges M. Parlow A. F. (2004). Generation of thyrotropin-releasing hormone receptor 1-deficient mice as an animal model of central hypothyroidism. Mol. Endocrinol. 18, 1450–1460. 10.1210/me.2004-0017 14988432

[B27] Romitti M. Tourneur A. De Faria Da Fonseca B. Doumont G. Gillotay P. Liao X. H. (2022). Transplantable human thyroid organoids generated from embryonic stem cells to rescue hypothyroidism. Nat. Commun. 13, 7057. 10.1038/s41467-022-34776-7 36396935 PMC9672394

[B28] Russell W. Harrison R. F. Smith N. Darzy K. Shalet S. Weetman A. P. (2008). Free triiodothyronine has a distinct circadian rhythm that is delayed but parallels thyrotropin levels. J. Clin. Endocrinol. Metab. 93, 2300–2306. 10.1210/jc.2007-2674 18364382

[B36] Singh J. Padgett R. A. (2009). Rates of in situ transcription and splicing in large human genes. Nat. Struct. Mol. Biol. 16 (11), 1128–1133. 10.1038/nsmb.1666 19820712 PMC2783620

[B29] Solovei I. (2010). Fluorescence *in situ* hybridization (FISH) on tissue cryosections. Methods Mol. Biol. 659, 71–82. 10.1007/978-1-60761-789-1_5 20809304

[B30] Trajkovic M. Visser T. J. Mittag J. Horn S. Lukas J. Darras V. M. (2007). Abnormal thyroid hormone metabolism in mice lacking the monocarboxylate transporter 8. J. Clin. Invest 117, 627–635. 10.1172/JCI28253 17318265 PMC1797602

[B31] Trajkovic-Arsic M. Muller J. Darras V. M. Groba C. Lee S. Weih D. (2010). Impact of monocarboxylate transporter-8 deficiency on the hypothalamus-pituitary-thyroid axis in mice. Endocrinology 151, 5053–5062. 10.1210/en.2010-0593 20702572

[B32] Van De Graaf S. A. Ris-Stalpers C. Pauws E. Mendive F. M. Targovnik H. M. De Vijlder J. J. (2001). Up to date with human thyroglobulin. J. Endocrinol. 170, 307–321. 10.1677/joe.0.1700307 11479128

[B33] Van Heuverswyn B. Streydio C. Brocas H. Refetoff S. Dumont J. Vassart G. (1984). Thyrotropin controls transcription of the thyroglobulin gene. Proc. Natl. Acad. Sci. U. S. A. 81, 5941–5945. 10.1073/pnas.81.19.5941 6592596 PMC391834

[B34] Vissel B. Choo K. H. (1989). Mouse major (gamma) satellite DNA is highly conserved and organized into extremely long tandem arrays: implications for recombination between nonhomologous chromosomes. Genomics 5, 407–414. 10.1016/0888-7543(89)90003-7 2613229

[B35] Walter J. Joffe B. Bolzer A. Albiez H. Benedetti P. A. Muller S. (2006). Towards many colors in FISH on 3D-preserved interphase nuclei. Cytogenet Genome Res. 114, 367–378. 10.1159/000094227 16954680

