# Corrigendum: The highly and perpetually upregulated thyroglobulin gene is a hallmark of functional thyrocytes

**DOI:** 10.3389/fcell.2025.1571466

**Published:** 2025-03-10

**Authors:** Simon Ullrich, Susanne Leidescher, Yana Feodorova, Katharina Thanisch, Jean-Baptiste Fini, Bernd Kaspers, Frank Weber, Boyka Markova, Dagmar Führer, Mirian Romitti, Stefan Krebs, Helmut Blum, Heinrich Leonhardt, Sabine Costagliola, Heike Heuer, Irina Solovei

**Affiliations:** ^1^ Biocenter, Ludwig Maximilians University Munich, Munich, Germany; ^2^ Department of Medical Biology, Medical University of Plovdiv, Division of Molecular and Regenerative Medicine, Research Institute at Medical University of Plovdiv, Plovdiv, Bulgaria; ^3^ Département Adaptations du Vivant (AVIV), Physiologie Moléculaire et Adaptation (PhyMA UMR 7221 CNRS), Muséum National d'Histoire Naturelle, CNRS, CP 32, Paris, France; ^4^ Department for Veterinary Sciences, Ludwig Maximilians University Munich, Planegg, Germany; ^5^ Department of General, Visceral and Transplantation Surgery, Section of Endocrine Surgery, University Duisburg-Essen, University Hospital Essen, Essen, Germany; ^6^ Department of Endocrinology, Diabetes and Metabolism, University Duisburg-Essen, University Hospital Essen, Essen, Germany; ^7^ IRIBHM ULB, Brussels, Belgium; ^8^ Laboratory for Functional Genome Analysis (LAFUGA), Gene Center, Ludwig Maximilians University Munich, Munich, Germany

**Keywords:** thyroglobulin gene, transcription loop, transcription, gene upregulation, thyroid hormones

In the published article, there was an error regarding the affiliation(s) for **Yana Feodorova**. As well as having affiliation **1**, they should also have “*Department of Medical Biology, Medical University of Plovdiv; Division of Molecular and Regenerative Medicine, Research Institute at Medical University of Plovdiv, Plovdiv, Bulgaria*.”

The authors apologize for this error and state that this does not change the scientific conclusions of the article in any way. The original article has been updated.

