# Supplementary material for: The highly and perpetually upregulated thyroglobulin gene is a hallmark of functional thyrocytes
Source: Front Cell Dev Biol. 2023 Oct 4;11:1265407. doi: 10.3389/fcell.2023.1265407 (PMC10582334; doi:10.3389/fcell.2023.1265407)
Supplement: Supplementary file 1 [file Presentation1.pdf]

Supplementary Material

<u>Supplementary Figure 1</u>. The *Tg* gene in cycling thyrocytes.

TLs remain detectable by RNA-FISH until early-to-mid prophase, disappearing only during late prophase. The gene remains silent during metaphase, anaphase and early telophase, and becomes active in early G1. Note the *Tg* TLs are present when most chromatin is already (prophase) or still (G1) condensed.

Top panel shows overlay of *Tg* signals (*green*) and DAPI (*red*); *mid panel* shows grey scale images of DAPI counterstain; *bottom panel* shows FISH signals with nuclear borders outlined with a *red line*.

Images are projections of *3-6 µm* confocal stacks. Scale bars: *5 µm*.

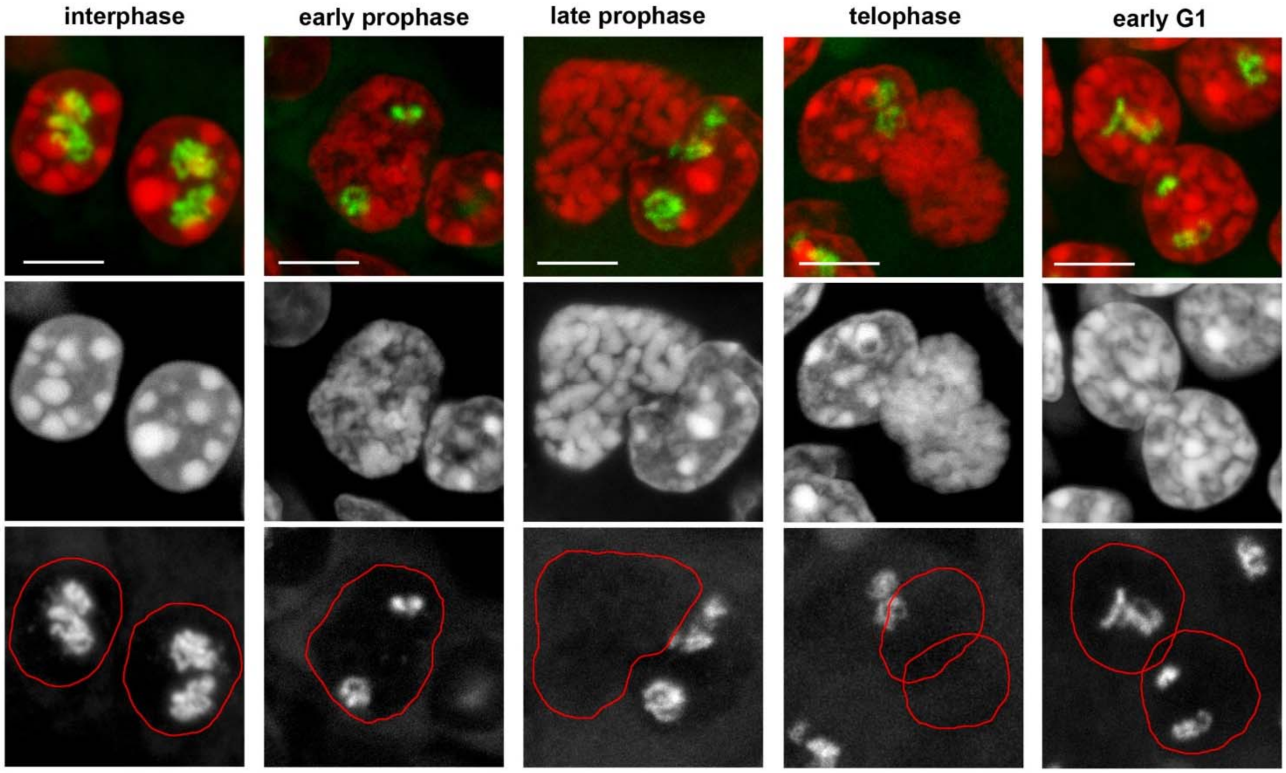

Supplementary Figure 2. The *Tg* TLs in mouse (A) and human (B) organoids.

RNA-FISH detecting *Tg* TLs (*green*) in nuclei (*red*) of thyroid organoids. Follicles formed in culture with matrigel (A1, B1) and thyrocytes within the follicles at a higher magnification (A2, B2, B3). For clarity, every RGB panel is supplemented by grey scale images of TLs.

Images are projections of *4.5 µm* confocal stacks. Scale bars: A1,B1, *10 µm*, A2, B2, B3, *5* µm.

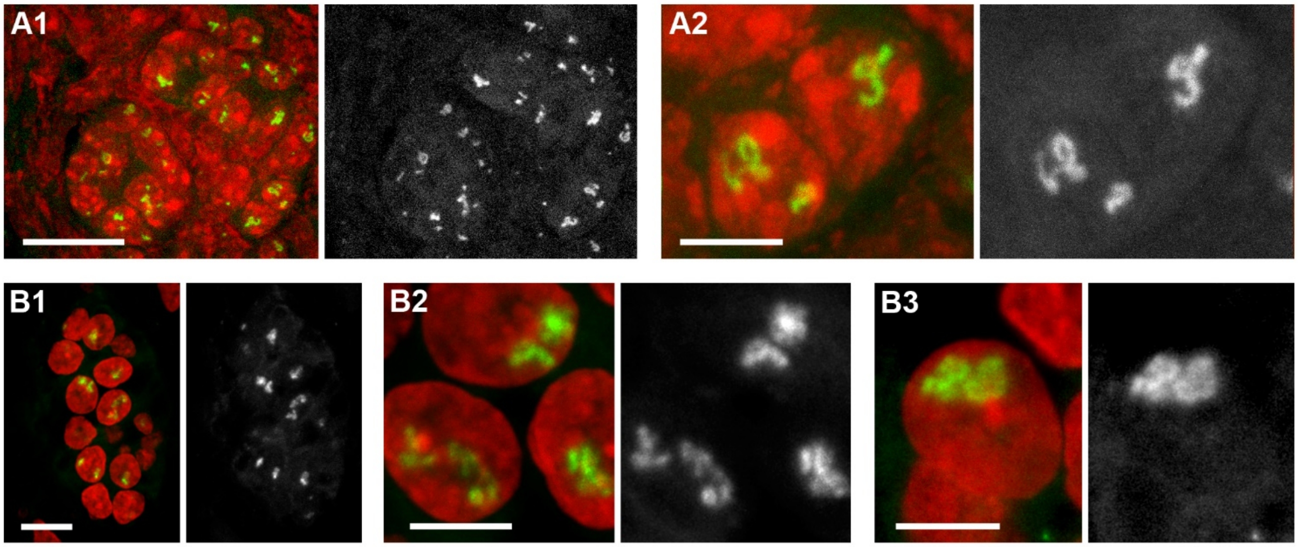

Supplementary Figure 3 (appended to FIGURE 4). *TG* genes expressed in grafted follicles manifest typical TL features.

(A) Human thyrocytes (*arrows*) grafted into mouse kidney can be unambiguously distinguished from mouse cells (*arrowheads*), because they possess brightly stained with DAPI chromocenters.

(B) In addition, grafted human thyrocytes can be distinguished by the presence of GFP fluorescence (for detail, see Rommiti et al 2022).

(C) Flanks of *TG* TLs are visibly separated. Left panels show grafted thyrocyte nuclei and 5' (*green*) and 3' (*red*) flanks; mid panels show flanks and TLs in *blue*; right panels show only TLs as grey scale images. Note that the nucleus on the right is from a tetraploid human thyrocyte with four *TG* TLs marked with arrows.

(D) *TG* TLs are decorated by nRNA transcripts undergoing co-transcriptional splicing. Three BAC probes highlighting introns sequentially label *TG* loops. The left panel shows the nucleus in *blue*, the 5' BAC in *green* and the 3' BAC in *red*; the next RGB panel shows all three BACs including the middle one (*blue*). For clarity, the other three panels show grey scale images of each BAC signal.

Schematics of genes and used BACs are shown above C and D panels. Nuclei are counterstained with DAPI (*blue*). Images are projections of *2-3 µm* confocal stacks. Scale bars: B, *25 µm*, A,C,D, *5 µm*.

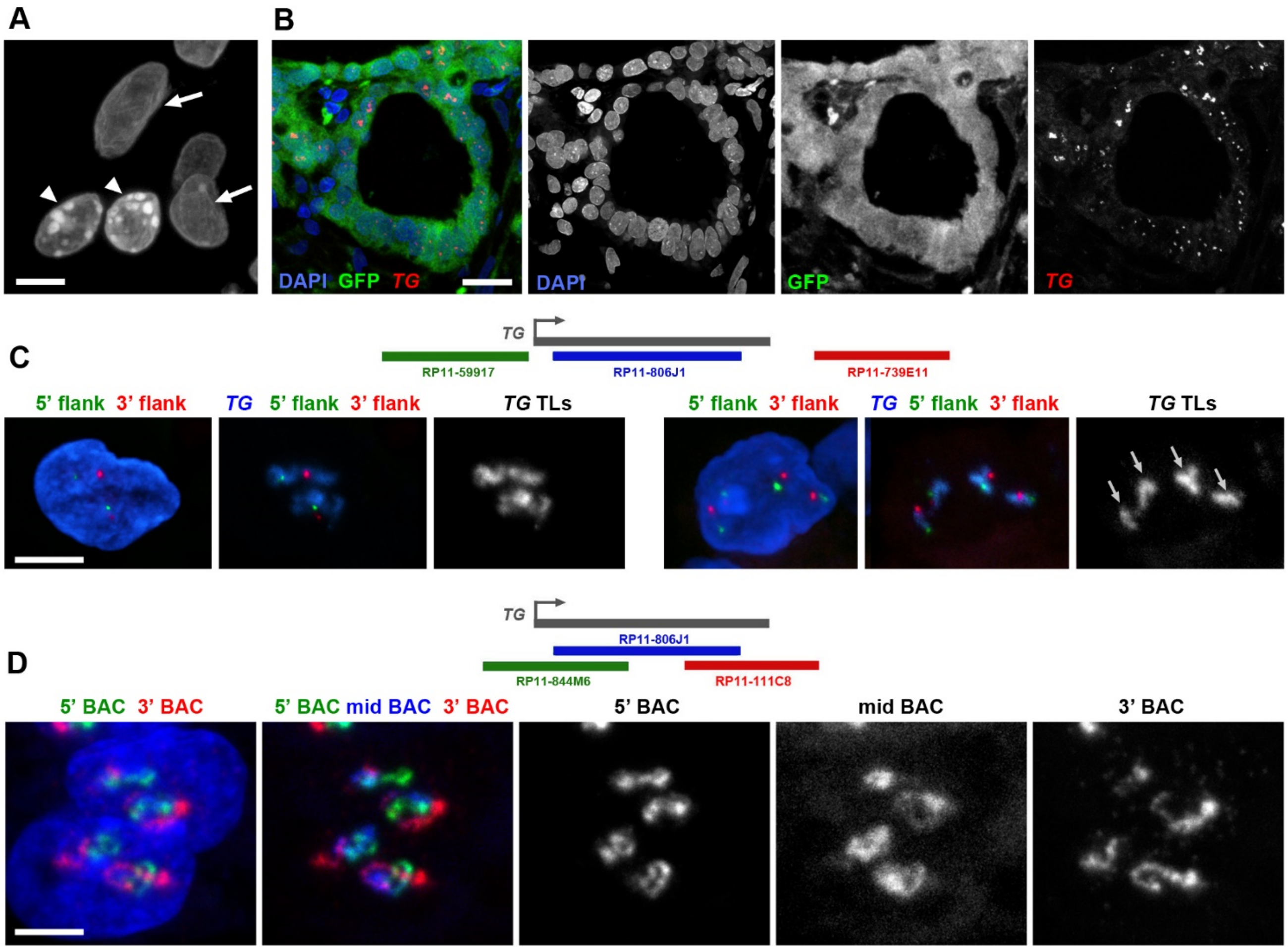

<u>Supplementary Figure 4</u>. Withdrawal of *Tg* TLs in primary thyrocyte culture.

(A, B) Freshly isolated follicle (A) and single thyrocytes (B) after RNA-FISH highlighting *Tg* TLs (*arrowheads*).

(C, D) After culturing for 1 (C) and 3 (D) days, mostly cubical thyrocytes flatten, enter the cell cycle and withdraw *Tg* TLs. Since the *Tg* genes become silenced and condensed, they can be detected only by DNA-FISH producing dot-like signals (*arrows*). Note the difference in the nuclear morphology of freshly isolated (B) and cultured for 3 days (D) cells.

DAPI, *red*; RNA and DNA signals, *green*; images are projections of *3-5 µm* confocal stacks; scale bars, *10 µm*

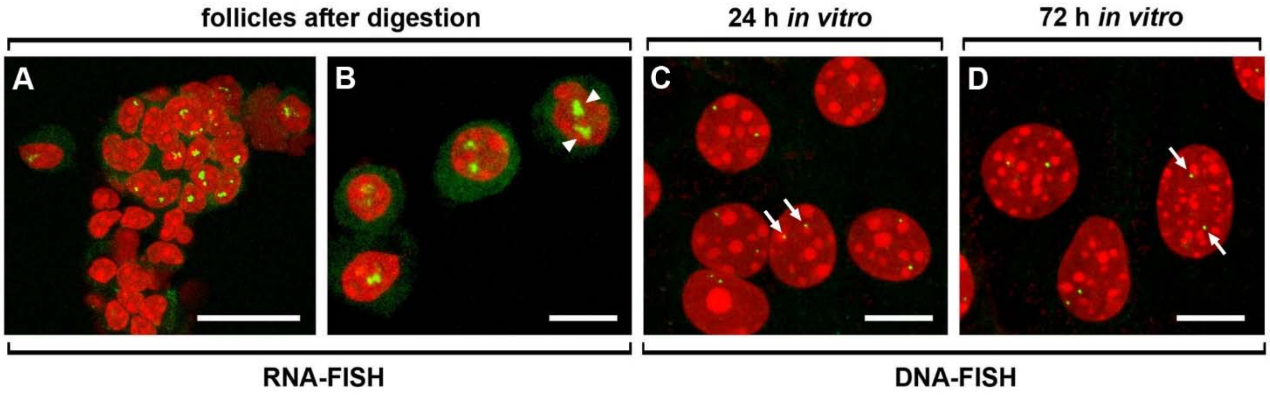

<u>Supplementary Figure 5</u> (appended to FIGURE 5). Follicles and exemplified thyrocyte nuclei after RNA-FISH detecting *Tg* TLs in mice models with decreased (*Trhr1*-KO) and increased (*Mct8*-KO and *Thrb*-KO) thyroidal TH production in comparison to control animals.

***Tg* TLs, *green*; DAPI, *red*; images are projections of 1-3 µm confocal stacks.**

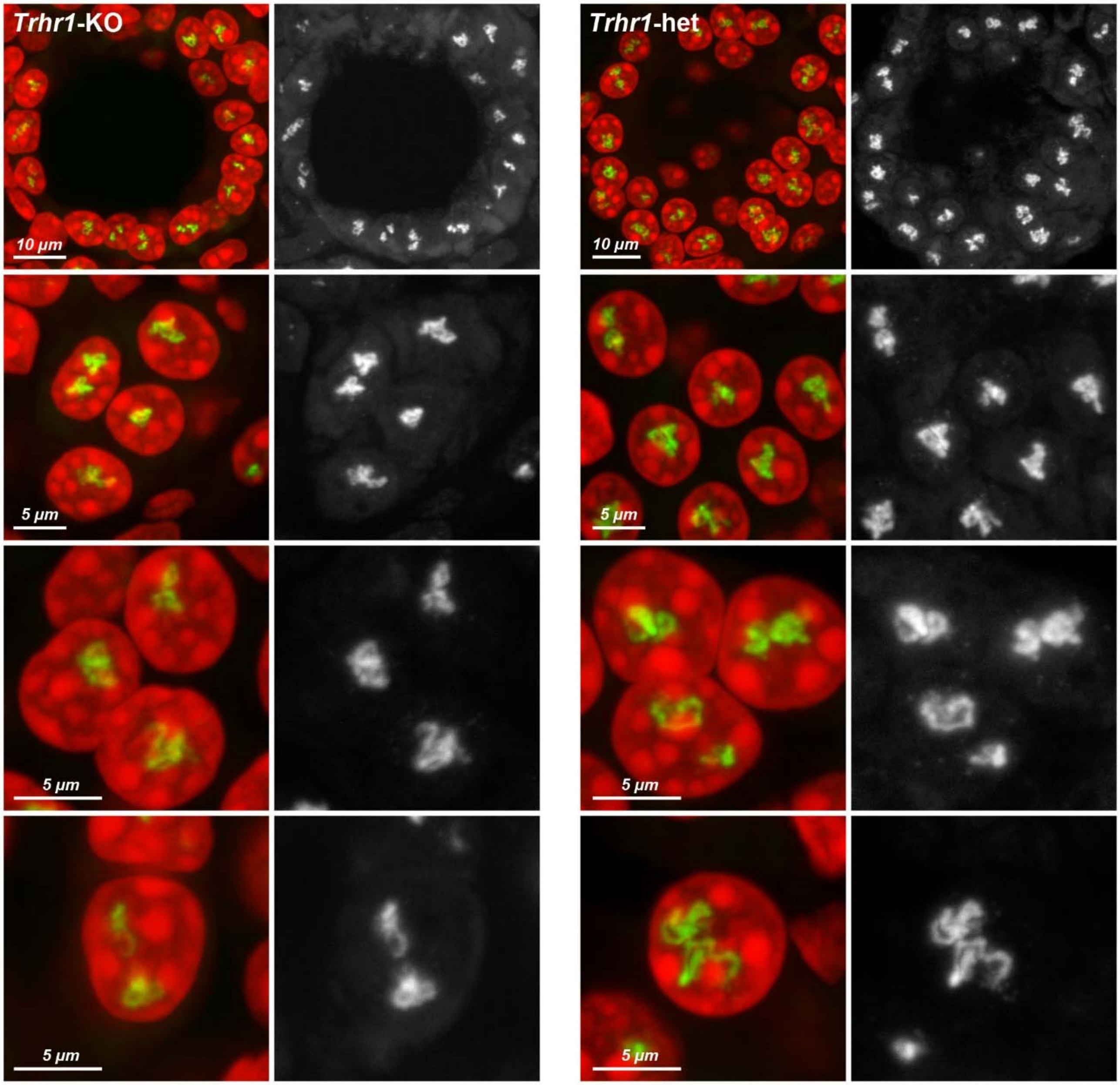

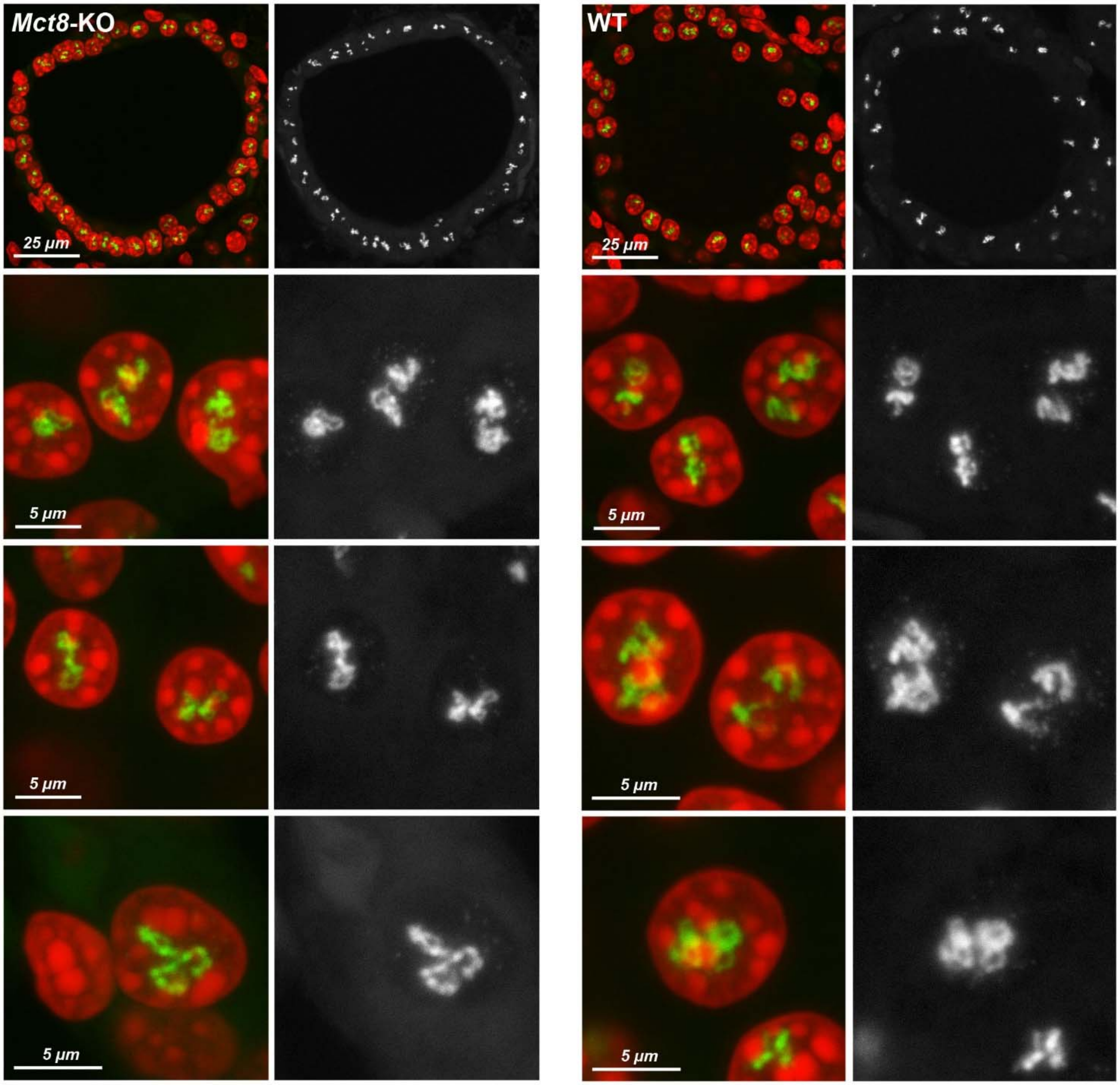
Mct8-KO
25 µm
5 µm
5 µm
5 µm
WT
25 µm
5 µm
5 µm
5 µm

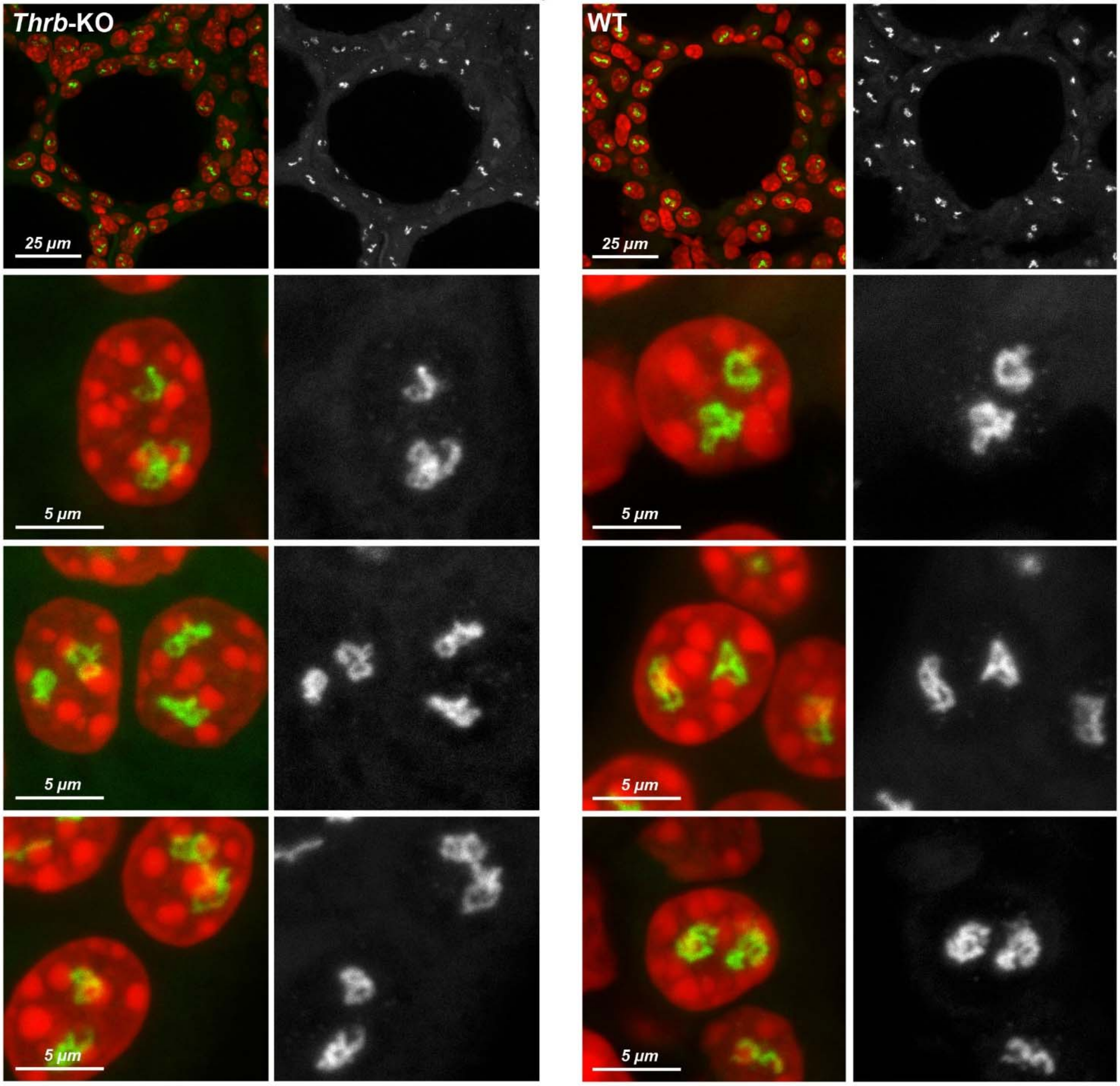

Thrb-KO
25 µm
5 µm
5 µm
5 µm
WT
25 µm
5 µm
5 µm
5 µm

<u>Supplementary Figure 6</u> (appended to FIGURE 6D). 20 examples of thyrocyte nuclei after RNA-FISH detecting *Tg* TLs at the four indicated time points used for the gene circadian activity test.

***Tg* TLs, *green*; DAPI, *red*; images are projections of 3 µm confocal stacks.**

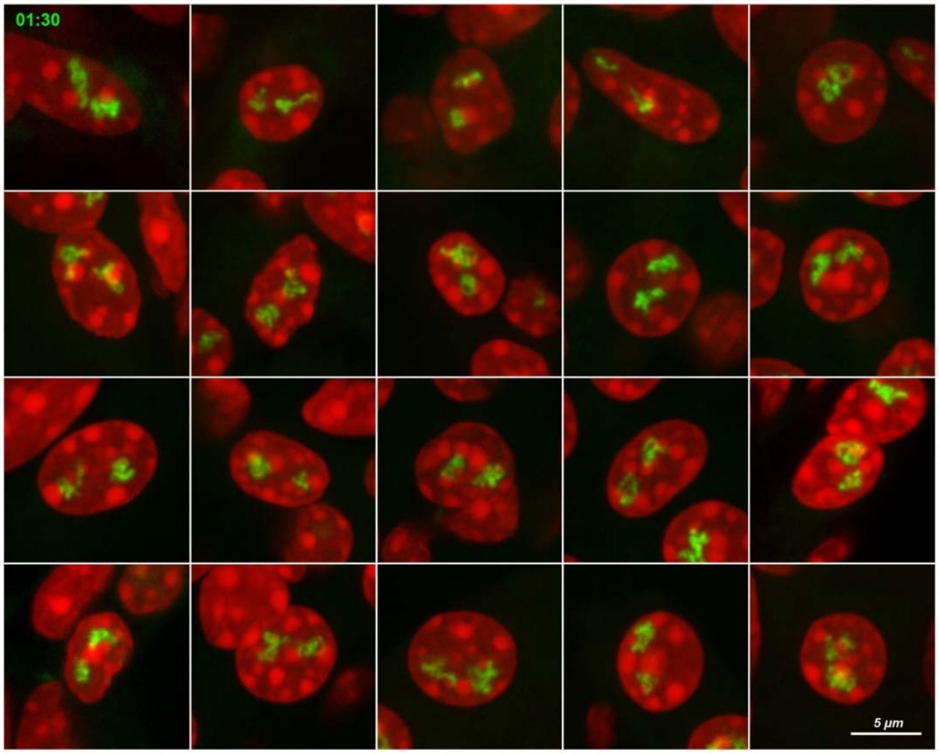

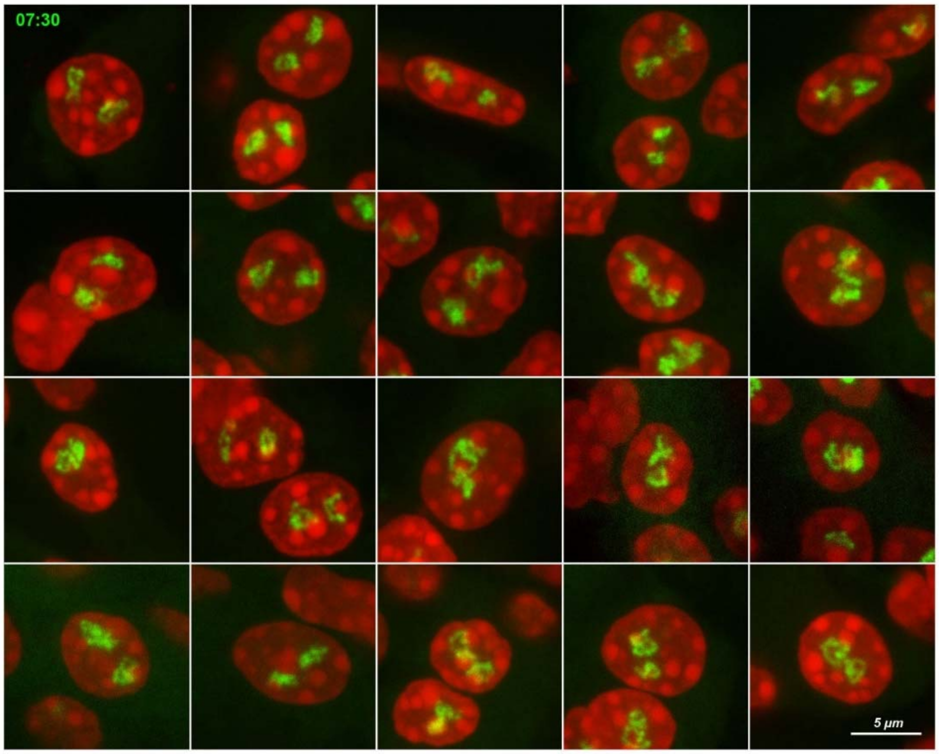

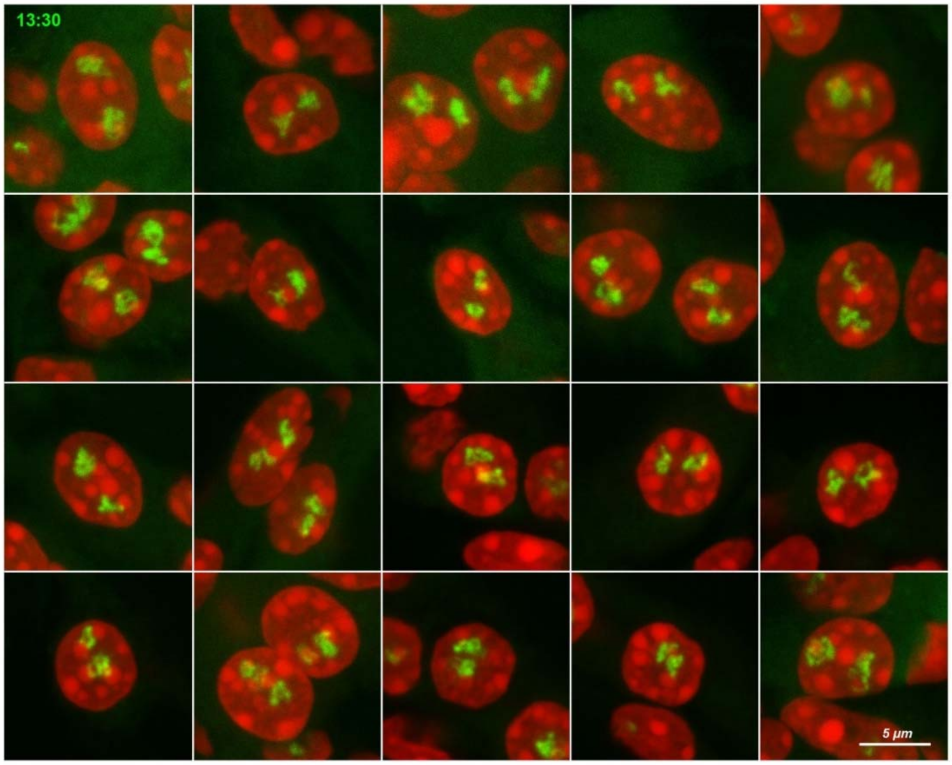
13:30
5 µm

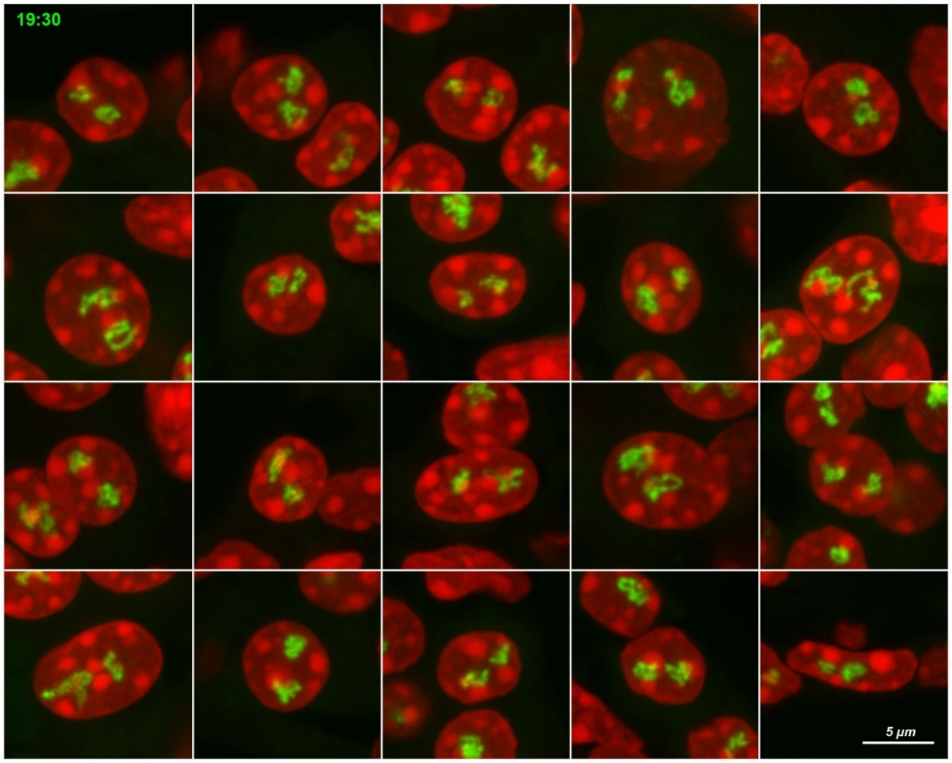
19:30
5 µm
